# Aplicação da Inteligência Artificial para Detecção, Caracterização e Estratificação de Risco de Pacientes com Insuficiência Tricúspide Grave

**DOI:** 10.36660/abc.20250743

**Published:** 2026-05-29

**Authors:** Luna Varela do Carmo, Auristela Isabel de Oliveira Ramos, Dorival Julio Della Togna, Eduardo da Silva Farias, Dimytri Alexandre de Alvim Siqueira

**Affiliations:** 1 Instituto Dante Pazzanese de Cardiologia São Paulo SP Brasil Instituto Dante Pazzanese de Cardiologia, São Paulo, SP – Brasil

**Keywords:** Insuficiência Cardíaca, Doenças das Valvas Cardíacas, Insuficiência da Valva Tricúspide, Inteligência Artificial

## Abstract

**Fundamento:**

A insuficiência tricúspide (IT) é prevalente e seu manejo permanece desafiador, com alta mortalidade na cirurgia isolada, indicação tardia à intervenção e recente expansão de terapias transcateter. No Brasil, há escassez de dados e de validação de ferramentas prognósticas.

**Objetivo:**

Investigar e definir o perfil clínico, laboratorial e ecocardiográfico de pacientes com IT grave a partir de variáveis coletadas por meio do treinamento de um modelo de inteligência artificial (IA).

**Métodos:**

Estudo observacional, unicêntrico, não intervencionista, de coorte retrospectiva, baseado em 71.911 laudos ecocardiográficos realizados entre 2021 e 2024. Um modelo de processamento de linguagem natural (PLN) foi desenvolvido em Python para identificar casos de IT grave, extrair variáveis e realizar o cálculo automatizado dos escores TRI-SCORE e EuroSCORE II, posteriormente revisados. Considerou-se valor de p bicaudal < 0,05 como estatisticamente significativo.

**Resultados:**

Foram identificados 803 pacientes com IT grave. A média de idade foi de 68,8 ± 13,5 anos, e 64,1% eram mulheres. Hipertensão arterial (71,7%) e fibrilação atrial (69,4%) foram as comorbidades mais prevalentes. A etiologia secundária predominou (96,8%), principalmente associada à valvopatia mitral (68,5%). O risco cirúrgico foi elevado (medianas: EuroSCORE II = 13,4%; TRI-SCORE = 7). A mortalidade foi de 17,1%. Na análise secundária de mortalidade, a regressão multivariada identificou preditores independentes, como TRI-SCORE em alto risco (relative risk [RR] 2,19; intervalo de confiança de 95% [IC 95%] 1,14-4,21) e creatinina elevada (RR 3,13; IC 95% 1,91-5,14).

**Conclusões:**

Na maior casuística brasileira de IT grave, construída com auxílio de IA, a doença demonstrou elevada complexidade clínica e prognóstica. A aplicação de PLN a grandes bases ecocardiográficas mostrou-se eficiente, podendo subsidiar estudos multicêntricos.

**Figure:**
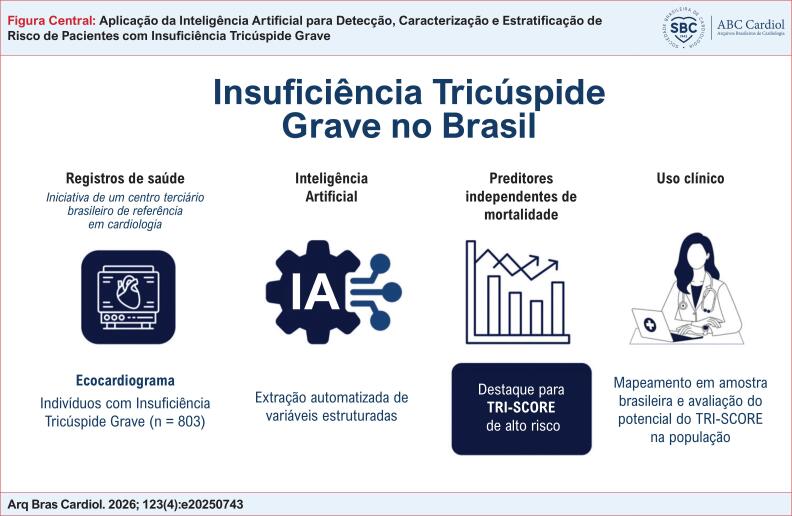


## Introdução

A insuficiência tricúspide (IT) é uma das valvopatias mais frequentes,^1^ alcançando uma prevalência de 4% em indivíduos > 75 anos^2^ e sendo mais comum no sexo feminino.^3^ Em graus moderados a graves, a IT está associada a um pior prognóstico, independentemente de comorbidades, níveis de pressão pulmonar e função do ventrículo direito (VD).^4^ Entre os fatores relacionados a desfechos desfavoráveis na IT grave, destacam-se idade avançada,^5,6^ disfunção do VD,^6–8^ hipertensão pulmonar,^7,9^ fibrilação atrial (FA),^5,10^ disfunção hepatorrenal^6^ e classes funcionais III-IV da classificação da *New York Heart Association* (NYHA).^11,12^

Em um estudo recente com 13.615 pacientes portadores de valvopatias aórtica, mitral e tricúspide, a mortalidade por todas as causas foi maior na presença de IT (*hazard ratio* [HR] 2,74; intervalo de confiança de 95% [IC 95%] 2,24-3,37), em comparação à valvopatia aórtica (HR 1,62; IC 95% 1,44-1,82) ou mitral (HR 1,25; IC 95% 1,09-1,44).^1^ Ademais, a IT significativa concomitante à estenose aórtica associa-se à mortalidade de 21,3% em 1 ano, independentemente do tratamento da estenose;^13^ no implante transcateter de valva aórtica, a presença prévia de IT significativa relaciona-se à maior mortalidade, e sua persistência após o procedimento associa-se à pior sobrevida.^14^

Quanto à estratificação de risco, os escores tradicionais de risco cirúrgico não foram desenvolvidos para prever desfechos de intervenção isolada da valva tricúspide. O TRI-SCORE foi proposto frente à expansão das terapias por cateter.^12^ Postula-se que o TRI-SCORE apresente desempenho superior na predição de mortalidade após reparo transcateter borda a borda da valva tricúspide, em comparação ao EuroSCORE II e ao STS-Score.^15^

No Brasil, persiste escassez de dados nacionais sobre prevalência, gravidade e prognóstico da IT. Em estudo transversal com 645 pacientes portadores de doença valvar reumática, observou-se taxa de 96,6% de IT funcional associada, sendo 12,8% dos casos classificados como moderados ou graves.^16^ Paralelamente, o avanço da ciência de dados tornou linguagens como Python amplamente utilizadas na medicina,^17^ com destaque para o processamento de linguagem natural (PLN) na interpretação automatizada de textos clínicos.

Os objetivos deste estudo foram definir o perfil clínico, laboratorial e ecocardiográfico de pacientes com IT grave a partir de variáveis extraídas por modelo de PLN, classificando-os segundo o EuroSCORE II e o TRI-SCORE e, adicionalmente, avaliar fatores associados à mortalidade.

## Métodos

### Delineamento e população do estudo

Estudo observacional, unicêntrico, não intervencionista, de coorte retrospectiva, incluindo pacientes com diagnóstico ecocardiográfico de IT grave no Instituto Dante Pazzanese de Cardiologia (IDPC), entre 2021 e 2024. Em 2025, foi realizada conferência retrospectiva dos registros institucionais para identificação de óbitos.

O estudo não teve como objetivo a estratificação etiológica em grupos mutuamente exclusivos, incluindo pacientes com IT grave independentemente da etiologia, da presença de doença valvar mitral e/ou aórtica concomitante ou de cirurgia cardíaca prévia, sendo essas associações descritas apenas para fins de caracterização.

### Critérios de elegibilidade

Foram elegíveis para inclusão indivíduos com idade > 18 anos, com diagnóstico ecocardiográfico de IT grave. Foram excluídos laudos de ecocardiograma transesofágico, exames transtorácicos referentes a cardiopatias congênitas e estudos com finalidade diagnóstica direcionada, que não permitissem avaliação global padronizada.

### Desenvolvimento do modelo de processamento de linguagem natural

**Figura 1:**
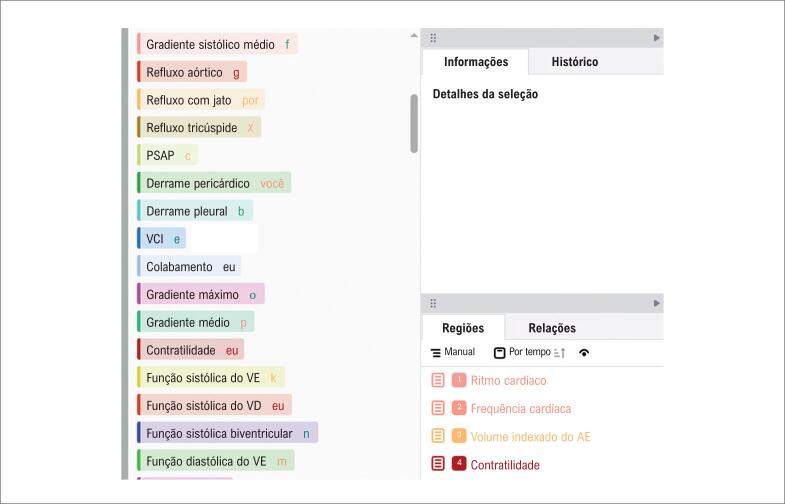
Recorte da plataforma Label Studio, com enfoque nos termos. AE: átrio esquerdo; PSAP: pressão sistólica da artéria pulmonar; VCI: veia cava inferior; VD: ventrículo direito; VE: ventrículo esquerdo.

Com base no modelo padronizado de laudos ecocardiográficos da instituição, foram definidas entidades nomeadas para identificação automatizada dos casos (Figuras 1 e 2). A estrutura textual típica dos laudos foi convertida em padrões operacionais para detecção, como no trecho "Valva tricúspide: (…). Ao Doppler, exibe refluxo de grau importante…", que foi mapeado para a combinação dos termos "refluxo tri_entity" e "importante", permitindo a identificação dos casos elegíveis.

Fonte: Elaboração própria.

**Figura 2:**
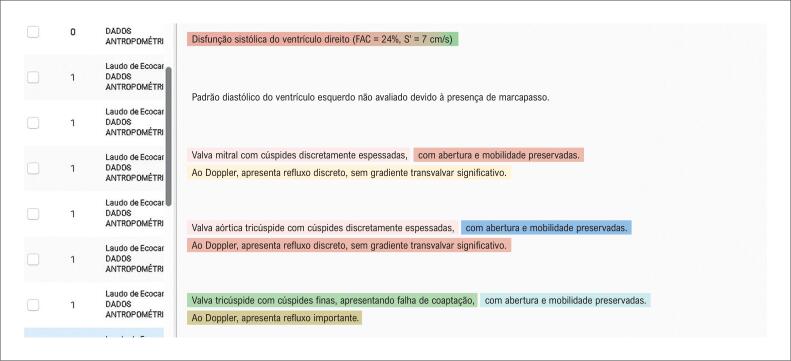
Recorte da plataforma Label Studio, com enfoque no corpo do laudo. FAC: *fractional area change*.

Fonte: Elaboração própria.

O modelo foi treinado a partir de 56 laudos previamente anotados manualmente na plataforma Label Studio, utilizando *pipeline* baseado em reconhecimento de entidades nomeadas e segmentação de sentenças (spaCy/Python). Após o treinamento, o modelo apresentou acurácia de 86% e foi aplicado para seleção dos pacientes e extração automatizada das variáveis de interesse.

Os termos ecocardiográficos utilizados no treinamento do modelo estão detalhados na Tabela B do Material Suplementar II. Abordagem semelhante foi empregada para extração de dados laboratoriais, a partir da plataforma institucional, e de informações clínicas, provenientes do prontuário eletrônico.

### Coleta e armazenamento de dados

Foram incluídos pacientes com pelo menos um laudo ecocardiográfico documentando IT grave no período estabelecido. Para análise das variáveis de interesse (Tabela A, Material Suplementar I), considerou-se o exame mais recente disponível.

Todos os casos identificados pelo modelo de IA foram submetidos à revisão manual, sendo confirmada a conformidade integral com os critérios de inclusão.

Os dados foram armazenados na plataforma REDCap e posteriormente exportados para o Microsoft Excel para organização, visualização e análise.

### Cálculo dos escores de risco

Os critérios e a padronização utilizados para o cálculo automatizado dos escores EuroSCORE II e TRI-SCORE encontram-se descritos nas Tabelas D e E do Material Suplementar II.

### Análise estatística

As variáveis categóricas foram descritas por frequências absolutas e relativas. As variáveis contínuas foram apresentadas como média ± desvio padrão quando apresentaram distribuição normal, ou como mediana e intervalo interquartil (Q1-Q3) quando não normalmente distribuídas. Os valores mínimo e máximo foram adicionalmente apresentados nas tabelas do Material Suplementar I.

Inicialmente, foram realizadas análises univariadas para comparação entre os grupos com e sem óbito. Para variáveis contínuas, utilizou-se o teste *U* de Mann-Whitney, e para variáveis categóricas, o teste do qui-quadrado em tabelas de contingência ou, quando apropriado, o teste exato de Fisher em tabelas 2 × 2 com frequência esperada < 5.

Diante da indisponibilidade do tempo exato até o óbito, o desfecho foi analisado em janela fixa. Foram estimados riscos relativos brutos e ajustados, com ICs 95%, por meio de regressão logística. A seleção de variáveis para o modelo multivariado seguiu estratégia *stepwise backward*, com critério de permanência de 10% e nível de significância de 5%.

As análises estatísticas foram realizadas no *software* Stata, versão 19.

Antes das análises, valores extremos foram sistematicamente revisados com base nos laudos ecocardiográficos originais. Valores confirmados como válidos foram mantidos, enquanto dados implausíveis ou sem variabilidade foram corrigidos ou excluídos.

### Considerações éticas

O estudo foi conduzido em conformidade com a Declaração de Helsinki e com a Resolução n° 196/96 do Conselho Nacional de Saúde. O protocolo foi aprovado pelo Comitê de Ética em Pesquisa do IDPC, por meio da Plataforma Brasil (CAAE 82634424.0.0000.5462; parecer n° 7.866.969).

## Resultados

### Construção da amostra

**Figura 3:**
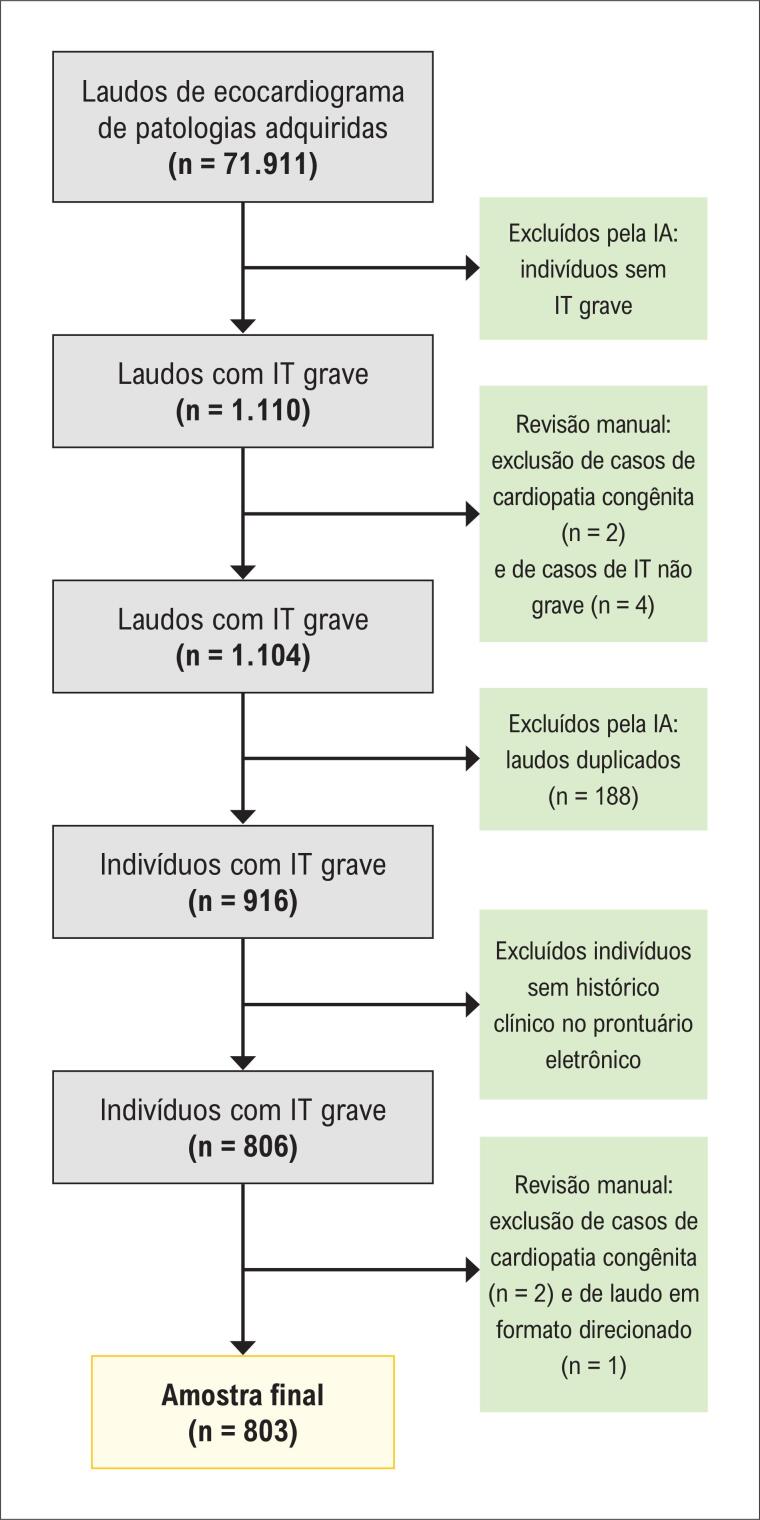
Fluxograma de construção da amostra. IA: inteligência artificial; IT: insuficiência tricúspide.

Entre 2021 e 2024, o instrumento identificou 71.911 laudos de ecocardiograma referentes a patologias adquiridas. Desses, 1.110 continham diagnóstico de IT grave. O fluxo de seleção da amostra está apresentado na Figura 3.

Fonte: Elaboração própria.

Após a aplicação dos critérios de elegibilidade e a revisão manual, 803 pacientes foram incluídos na análise final. Desses, 138 evoluíram a óbito na instituição, conforme verificação retrospectiva dos prontuários realizada em 2025.

Foi realizada a caracterização basal da amostra, incluindo variáveis clínicas, laboratoriais e ecocardiográficas da população geral, além de análises univariada e multivariada direcionadas ao desfecho de mortalidade.

### População geral do estudo

As características clínicas, laboratoriais e ecocardiográficas da população encontram-se detalhadas nas Tabelas B, C e D do Material Suplementar I. A coorte incluiu 803 pacientes com IT grave, composta predominantemente por mulheres idosas, com idade média de 68,8 ± 13,5 anos, e ampla variação do índice de massa corporal.

A maioria dos pacientes referiu nunca ter fumado, e o etilismo foi relatado por uma minoria da amostra.

### Características clínicas

A população apresentou elevada carga de comorbidades, com destaque para hipertensão arterial sistêmica, dislipidemia, diabetes mellitus e doença renal crônica (Tabela 1). A FA esteve presente em aproximadamente dois terços da coorte.

**Tabela 1:** Características basais da amostra

Característica		Amostra total (n = 803)
Dados demográficos		
Idade, anos†		68,8 ± 13,5
Sexo feminino, n (%)		515 (64,1)
IMC, kg/m^2^†		26,7 ± 5,4
**Hábitos de vida, n (%)**		
**Tabagismo**		
	Não tabagista	512 (63,8)
	Ex-tabagista	251 (31,3)
	Tabagismo atual	40 (5,0)
**Etilismo**		
	Não etilista	688 (85,7)
	Etilista	115 (14,3)
**Escores de risco**		
**EuroSCORE II**‡		13,4 (8,1-21,3)
	Baixo, n (%)	43 (5,4)
	Intermediário, n (%)	155 (19,3)
	Alto, n (%)	605 (75,3)
**TRI-SCORE odds**‡		7 (6-9)
	Baixo, n (%)	51 (6,3)
	Intermediário, n (%)	145 (18,1)
	Alto, n (%)	607 (75,6)
**Características clínicas, n (%)**		
Classes III-IV da NYHA		127 (15,9)
Edema		62 (7,7)
HAS		576 (71,7)
DM em uso de insulina		42 (5,2)
DM		199 (24,8)
FA		509 (69,4)
Doença pulmonar		132 (16,4)
Valvopatia mitral		550 (68,5)
Valvopatia aórtica		183 (22,8)
Uso de iECA		271 (33,8)
Uso de BRA		274 (34,1)
**Tratamento, n (%)**		
**Uso de furosemida**		610 (76,0)
	Dose > 125 mg/dia	69 (8,6)
**Uso de anticoagulantes**		512 (63,8)
	Varfarina	268 (33,4)
	Rivaroxabana	244 (30,4)
**Histórico cardiovascular, n (%)**		
Cirurgia cardíaca prévia		235 (29,3)
DCEI		123 (15,3)
Prótese mitral		174 (21,7)
Prótese aórtica		106 (13,2)
Plastia tricúspide		34 (4,2)

† Valores expressos em média ± desvio padrão.

‡ Valores expressos em mediana (intervalo interquartil). BRA: bloqueadores do receptor de angiotensina; DCEI: dispositivo cardíaco eletrônico implantável; DM: diabetes mellitus; HAS: hipertensão arterial sistêmica; FA: fibrilação atrial; iECA: inibidores da enzima conversora da angiotensina; IMC: índice de massa corporal; NYHA: New York Heart Association. Faixas do EuroSCORE II: baixo risco (< 4%); risco intermediário (4%-8%); alto risco (> 8%). Faixas do TRI-SCORE: baixo risco (≤ 3 pontos); risco intermediário (4-5 pontos); alto risco (≥ 6 pontos).

Fonte: Elaboração própria.

A hipertensão pulmonar foi observada na maioria dos pacientes avaliados, com proporção relevante apresentando níveis elevados de pressão sistólica da artéria pulmonar (PSAP). Do ponto de vista clínico, predominaram as classes funcionais I-II da classificação da NYHA. Além disso, parte dos pacientes apresentava dispositivos cardíacos eletrônicos implantáveis e sinais clínicos de congestão periférica (Tabela 1).

### Etiologia da insuficiência tricúspide

A forma secundária de IT foi predominante (777/803; 96,8%), em consonância com o fenótipo funcional da coorte, enquanto a forma primária foi identificada em 26 pacientes (3,2%).

Entre os casos secundários, observou-se associação frequente com valvopatias esquerdas, principalmente mitral (68,5%) e, em menor proporção, aórtica (22,8%). Nos casos primários com etiologia definida (n = 22), predominaram as etiologias reumáticas, seguidas por rotura de cordoalha, anomalia de Ebstein, *cleft* da valva e síndrome carcinoide.

### Medicações em uso

Quanto ao tratamento medicamentoso, a maioria dos pacientes fazia uso de inibidores da enzima conversora da angiotensina ou bloqueadores do receptor de angiotensina. A anticoagulação oral, com varfarina ou rivaroxabana, foi amplamente empregada, embora uma parcela menor da coorte não tenha utilizado esse tipo de terapia.

O uso de diuréticos de alça foi também frequente, com predominância de furosemida, incluindo uma fração de pacientes em doses elevadas. Por outro lado, o uso de insulina foi pouco prevalente.

### Procedimentos prévios e estratificação de risco

Entre os 803 pacientes incluídos, 235 (29,3%) apresentavam histórico de cirurgia cardíaca prévia, sendo mais frequente a presença de prótese mitral (biológica ou mecânica), seguida por prótese aórtica e plastia tricúspide prévia.

A estratificação de risco evidenciou uma coorte predominantemente de alto risco cirúrgico segundo o EuroSCORE II, com valores elevados na maioria dos pacientes e predominância da categoria de alto risco. Resultados semelhantes foram observados com o TRI-SCORE, tanto na versão simples quanto na forma padronizada por *odds*, ambas classificando aproximadamente três quartos da população como de alto risco (Tabela 1).

### Características laboratoriais

Houve ampla variabilidade nos parâmetros laboratoriais, o que reflete diferentes graus de comprometimento sistêmico na coorte (Tabela 2).

**Tabela 2:** Variáveis laboratoriais

Variável (unidade)	Amostra
Hematócrito, %†	37,5 (33,1-41,6)
Leucócitos, 10^3^/μ†	6,8 (5,4-8,7)
Plaquetas, células/μ†	189.000 (147.000-236.000)
Creatinina, mg/dl†	1,3 (1,0-1,7)
TFG, ml/min/1,73 m^2^†	52 (35-72)
NT-proBNP, pg/ml†	2.517 (905-9.060)
Bilirrubinas totais aumentadas, n (%)	135 (17,3)

† Valores expressos em mediana (intervalo interquartil). TFG: taxa de filtração glomerular; NT-proBNP: fragmento N-terminal do pró-peptídeo natriurético tipo B.

Fonte: Elaboração própria.

A Tabela 2 apresenta os valores medianos de hematócrito, leucócitos, plaquetas, creatinina, taxa de filtração glomerular e fragmento N-terminal do pró-peptídeo natriurético tipo B (NT-proBNP). Além disso, níveis elevados de bilirrubinas totais foram identificados em parte dos pacientes.

### Características ecocardiográficas

O detalhamento completo encontra-se na Tabela 3. Observou-se dilatação significativa do átrio esquerdo, associada a remodelamento do ventrículo esquerdo (VE) e ampla variabilidade da função sistólica, com fração de ejeção reduzida em mais da metade da coorte.

**Tabela 3:** Variáveis ecocardiográficas

Variável	Amostra
Etiologia secundária, n (%)	777 (96,8)
VCI, mm†	23 (20-27)
Colabamento < 50%, n (%)	536 (67,6)
Derrame pericárdico, n (%)	143 (17,8)
PSAP, mmHg†	53 (45-65)
Disfunção do VD, n (%)	436 (54,3)
FEVE, %†	48 (31-58)
Disfunção importante do VE, n (%)	188 (23,4)
Diâmetro diastólico final do VE, mm†	54 (48-60)
Diâmetro sistólico final do VE, mm†	38 (31-48)

† Valores expressos em mediana (intervalo interquartil). FEVE: fração de ejeção do ventrículo esquerdo; PSAP: pressão sistólica da artéria pulmonar; VCI: veia cava inferior; VD: ventrículo direito; VE: ventrículo esquerdo.

Fonte: Elaboração própria.

A função sistólica do VD encontrava-se comprometida em proporção semelhante. A PSAP apresentou ampla variação, com valores elevados na maioria dos pacientes (mediana de 53 mmHg), frequentemente acompanhados de dilatação da veia cava inferior (VCI) e redução do colabamento inspiratório.

Derrame pericárdico foi identificado em parte dos casos. Do ponto de vista morfológico, observou-se espessamento valvar frequente nas valvas tricúspide, mitral e aórtica.

No ecocardiograma mais recente disponível, a maioria dos pacientes manteve IT grave. Entre aqueles que apresentaram redução da gravidade, parte havia sido submetida a intervenções cirúrgicas, incluindo plastia tricúspide, transplante cardíaco ou cirurgia valvar esquerda, enquanto os demais permaneceram em tratamento conservador.

### Análise de regressão multivariada

As variáveis identificadas na análise univariada ou consideradas clinicamente relevantes foram incluídas no modelo de regressão multivariada.

No modelo final (n = 763), ajustado por regressão logística com seleção *stepwise backward*, permaneceram como preditores independentes de mortalidade: perfil de alto risco cirúrgico pelo TRI-SCORE, alterações laboratoriais indicativas de disfunção orgânica (hematócrito reduzido, leucocitose, plaquetopenia e creatinina elevada) e achados ecocardiográficos associados à congestão sistêmica, como dilatação da VCI e derrame pericárdico.

Entre as variáveis estruturais, o espessamento valvar aórtico também se manteve associado ao desfecho. Por outro lado, o uso de rivaroxabana apresentou associação independente com menor probabilidade de mortalidade (Tabela 4).

**Tabela 4:** Análise multivariada

Variável	Categoria	RR	IC 95%	Valor de p
TRI-SCORE	Baixo + intermediário	1,00	Referência	—
	Alto	2,19	1,14-4,21	0,019
Anticoagulante	Não	1,00	Referência	—
	Varfarina	0,62	0,37-1,04	0,072
	Rivaroxabana	0,44	0,26-0,76	0,003
Hematócrito	Normal	1,00	Referência	—
	Alterado	2,30	1,32-4,01	0,003
Leucócitos	Normal	1,00	Referência	—
	Alterado	3,08	1,96-4,83	< 0,001
Plaquetas	Normal	1,00	Referência	—
	Alterado	2,25	1,45-3,49	< 0,001
Creatinina	Normal	1,00	Referência	—
	Alterado	3,13	1,91-5,14	< 0,001
Valva aórtica espessada	Não	1,00	Referência	—
	Sim	1,98	1,09-3,61	0,025
Derrame pericárdico	Não	1,00	Referência	—
	Sim	1,71	1,03-2,82	0,038
Veia cava inferior	Normal	1,00	Referência	—
	Alterado	2,44	1,37-4,35	0,002

IC: intervalo de confiança; RR: *relative risk*.

Fonte: Elaboração própria.

## Discussão

### Achados principais e comparação com a literatura

A presente coorte, composta por 803 pacientes com IT grave, representa, até o momento, a maior casuística nacional sobre o tema. A construção da amostra foi viabilizada por meio da aplicação de inteligência artificial (IA), utilizando um modelo de PLN desenvolvido especificamente para este estudo. Esse modelo permitiu a identificação automatizada e padronizada de laudos compatíveis com IT grave em um universo superior a 70.000 exames ecocardiográficos realizados ao longo de 4 anos na instituição.

Essa estratégia possibilitou maior eficiência na triagem dos casos, redução de vieses associados à extração manual de dados e aumento da reprodutibilidade do processo, por meio da seleção sistematizada dos pacientes incluídos. Todos os casos identificados pelo modelo como IT grave foram confirmados após revisão manual dos laudos ecocardiográficos, reforçando a acurácia da abordagem empregada.

A população estudada caracterizou-se por idade avançada (média de 68,8 ± 13,5 anos) e predominância do sexo feminino (64,1%), perfil compatível com registros internacionais, como o *Framingham Heart Study*,^3^ que demonstrou maior prevalência de IT moderada a grave em mulheres idosas, principalmente na faixa etária entre 70-83 anos. Observou-se elevada prevalência de comorbidades, incluindo hipertensão arterial sistêmica, dislipidemia, doença renal crônica e doença arterial coronariana, condições frequentes nessa faixa etária e associadas a maior risco cardiovascular.

A FA esteve presente em 69,4% dos pacientes, enquanto aproximadamente 43% apresentaram PSAP ≥ 55 mmHg. A coexistência desses achados reflete mecanismos fisiopatológicos relevantes na IT, incluindo dilatação atrial, hiperfluxo e desenvolvimento de IT funcional atrial, além de possíveis manifestações de doença em estágio mais avançado.

A prevalência de FA observada foi inferior à reportada em estudos clínicos como TRILUMINATE^18^ (90%) e TRISCEND II^19^ (96,1%), porém semelhante à descrita no TRIGISTRY^20^ (66%). Cerca de 63% da população utilizava anticoagulantes orais, proporção compatível com a prevalência de FA na coorte.

A maioria dos pacientes encontrava-se em classes funcionais I-II da NYHA (84%), evidenciando dissociação entre gravidade anatômica da IT e intensidade dos sintomas. Apesar disso, 76% faziam uso de diuréticos, refletindo manejo clínico da congestão sistêmica. Manifestações clínicas mais avançadas (p.ex., dispneia em classes III-IV [15,9%], uso de furosemida em doses superiores a 125 mg/dia [8,6%] e edema de membros inferiores [7,7%]) foram menos frequentes, características que, em geral, orientam a indicação de intervenção.

Assim, em estudos contemporâneos que comparam estratégias de tratamento percutâneo, os critérios de inclusão tendem a privilegiar pacientes com sintomatologia refratária, o que resulta em maior proporção de indivíduos em classes funcionais mais avançadas. Como reflexo desse perfil, a taxa de uso de diuréticos em nossa coorte foi inferior à observada em outras séries com portadores de IT grave.^18,20^ Adicionalmente, não se pode excluir a possibilidade de subvalorização clínica dos sintomas ou de imprecisões nos registros médicos, fatores que podem contribuir para a discrepância entre o estado funcional reportado e a gravidade da disfunção tricúspide.

Cabe destacar que a análise do tratamento medicamentoso teve como foco o uso de diuréticos de alça como marcador indireto de manejo da congestão sistêmica, não tendo sido objetivo do estudo a comparação entre diferentes classes de diuréticos.

A etiologia secundária ou funcional da IT foi predominante nesta coorte (97%). A elevada prevalência de valvopatias esquerdas significativas associadas, especialmente a doença mitral (68,5%), está em concordância com pesquisas anteriores,^18–21^ que descrevem associação frequente entre insuficiência mitral grave e IT grave, acometendo cerca de 30%-50% desses pacientes.

Aproximadamente um terço da população já havia sido submetido à cirurgia cardíaca, proporção discretamente inferior à relatada em outras séries^18,22^ (29,3% vs 37% e 40%, respectivamente). A taxa de intervenção valvar prévia envolvendo a valva tricúspide, de forma isolada ou concomitante, foi baixa (4%), achado consistente com dados de pesquisa prévia.^23^

A proporção de etiologia secundária observada supera as estimativas tradicionais, que indicam prevalência aproximada de 80% de IT funcional, 5%-10% de IT primária e cerca de 10% associada a dispositivos cardíacos eletrônicos implantáveis (DCEI).^4,24^ Esse achado pode ser parcialmente explicado pelos critérios de inclusão adotados, que contemplaram exclusivamente valvopatias adquiridas e excluíram formas congênitas, limitando a identificação de etiologias primárias, as quais corresponderam a 3,2% dos casos. Aproximadamente 15% dos pacientes eram portadores de DCEI.

Os achados laboratoriais refletem o impacto sistêmico da IT grave, evidenciando alterações compatíveis com congestão venosa crônica. Reduções nos níveis de hematócrito e plaquetas, associadas à elevação de creatinina e bilirrubinas, sugerem comprometimento multissistêmico. O aumento de marcadores neuro-hormonais, como o NT-proBNP, reforça a presença de sobrecarga hemodinâmica persistente e ativação compensatória, características de pacientes com disfunção do VD e hipertensão pulmonar associadas.

Após a estratificação de risco pelos escores EuroSCORE II e TRI-SCORE, mais de 75% dos pacientes foram classificados como de alto risco cirúrgico, evidenciando o perfil clínico complexo da coorte. Esse cenário sugere que, conforme diretrizes contemporâneas,^25^ abordagens transcateter podem representar alternativa terapêutica relevante, quando anatomicamente viáveis e disponíveis. Além disso, a proporção de pacientes classificados como alto risco pelo TRI-SCORE foi substancialmente superior à descrita por Dreyfus et al.^20^ (75% vs 35%), o que reforça a maior gravidade clínica desta população.

Os achados ecocardiográficos evidenciam alterações estruturais e funcionais compatíveis com remodelamento cardíaco biventricular. Os diâmetros sistólico e diastólico finais do VE apresentaram valores medianos limítrofes a elevados para mulheres, mas dentro da normalidade para homens, conforme os pontos de corte descritos no material suplementar. O diâmetro diastólico do VE foi semelhante ao observado nos estudos TRISCEND II^19^ e TRILUMINATE^18^(≈ 50-55 mm), sugerindo remodelamento discreto do VE.

A fração de ejeção do VE (mediana de 48%) foi próxima aos valores descritos no registro TRIGISTRY^20^ e no estudo EVOQUE TTVR^22^ (≈ 51%-54%), indicando disfunção sistólica discreta. Destaca-se que 23,4% dos pacientes apresentavam disfunção importante do VE, possivelmente relacionada a valvopatias prévias, interação interventricular e/ou hipertensão pulmonar.

A disfunção do VD esteve presente em 54,3% dos pacientes, valor compatível com a faixa reportada em coortes multicêntricas (45%-60%), reforçando o papel central do remodelamento das câmaras direitas na fisiopatologia da IT grave. Além disso, os valores elevados de PSAP (≈ 55 mmHg) corroboram a presença de sobrecarga pressórica crônica, fator determinante para a progressão da disfunção do VD e perpetuação da IT.

Em aproximadamente 15% dos pacientes, observou-se variação na quantificação da IT entre o ecocardiograma-índice, utilizado para inclusão no estudo, e o exame mais recente, a partir do qual foram extraídas as variáveis. Nesses casos, o exame inicial evidenciava IT grave, enquanto avaliações subsequentes demonstraram redução do grau de IT. Entre os indivíduos que apresentaram essa variação, 41,4% foram submetidos à intervenção cirúrgica, incluindo transplante cardíaco ou cirurgia do lado esquerdo, com ou sem abordagem concomitante da valva tricúspide. Por outro lado, mais da metade dos pacientes permaneceu em tratamento clínico, sugerindo que flutuações hemodinâmicas e mecanismos compensatórios podem modular a gravidade da IT ao longo do tempo.

Entre o período de inclusão dos exames (2021-2024) e o congelamento da base (julho de 2025), quando foi realizada revisão retrospectiva dos registros institucionais para identificação dos desfechos, 138 pacientes (17,1%) evoluíram a óbito. Essa proporção corresponde à mortalidade acumulada dentro da janela fixa de observação adotada no estudo, não refletindo o tempo individual até o evento, uma vez que não houve seguimento longitudinal sistemático. A taxa de mortalidade observada foi expressiva e semelhante à descrita em pesquisa prévia,^26^ que apontou mortalidade aproximada de 20% em pacientes com IT grave em um período mediano de seguimento de 2,7 anos.

Para identificar fatores associados à mortalidade, foi realizada uma análise exploratória que incluiu variáveis clínicas, laboratoriais e ecocardiográficas. Na análise multivariada, permaneceram como preditores independentes de mortalidade: TRI-SCORE elevado, alterações laboratoriais (p.ex., hematócrito reduzido, leucocitose, plaquetopenia e creatinina elevada), espessamento da valva aórtica, dilatação da VCI, presença de derrame pericárdico e ausência de uso de rivaroxabana.

Os parâmetros laboratoriais mantiveram associação independente com o desfecho, corroborando seu valor prognóstico. O hematócrito reduzido pode refletir anemia de doença crônica; a plaquetopenia pode estar relacionada a hiperesplenismo ou congestão portal; a leucocitose pode indicar processos inflamatórios ou infecciosos intermitentes; e a creatinina elevada sugere disfunção renal, frequentemente de caráter congestivo ou multifatorial. A persistência dessas variáveis no modelo ajustado reforça a importância da avaliação laboratorial rotineira como ferramenta prognóstica, conforme descrito em estudos recentes e incorporado a escores contemporâneos, como o *Isolated Tricuspid Valve Surgery Risk Calculator* da *Society of Thoracic Surgeons* (STS).^27^

Entre as variáveis ecocardiográficas, a dilatação da VCI e o derrame pericárdico mantiveram associação significativa com mortalidade, reforçando o papel da congestão venosa sistêmica e do remodelamento das câmaras direitas como marcadores de doença avançada.^28^ O aumento do diâmetro da VCI constitui marcador consolidado de elevação da pressão atrial direita e sobrecarga volêmica, frequentemente descrito em casos graves de IT.^29,30^ O derrame pericárdico, por sua vez, pode refletir descompensação hemodinâmica e elevação das pressões intracardíacas, sendo frequentemente associado à hipertensão pulmonar e à disfunção do VD, mesmo quando de pequena magnitude.^31^

O espessamento da valva aórtica também permaneceu como preditor independente, sugerindo que esse achado, embora não diretamente relacionado à valva tricúspide, possa refletir doença cardiovascular sistêmica e degeneração valvar difusa, associadas ao envelhecimento, à rigidez arterial e à maior carga aterosclerótica, características de pacientes em estágios mais avançados da doença.

O uso de rivaroxabana apresentou associação independente com menor mortalidade. Esse efeito pode estar relacionado à redução de eventos tromboembólicos e à possível diminuição da microtrombose pulmonar, com consequente menor sobrecarga do VD. Adicionalmente, características farmacológicas favoráveis, como farmacocinética previsível e ausência de variabilidade da razão normalizada internacional (RNI), podem ter contribuído para a consistência do efeito observado. Entretanto, não se pode excluir a influência de viés de canalização terapêutica, uma vez que anticoagulantes diretos tendem a ser prescritos em pacientes com FA não valvar e perfil clínico menos grave.

O TRI-SCORE destacou-se como o preditor mais robusto no modelo multivariado, mesmo após ajuste para múltiplas covariáveis, evidenciando seu caráter integrado e multidimensional. No modelo final, o escore incorporou o efeito das variáveis que o compõem, evitando a colinearidade e se consolidando como marcador independente de risco.

### Implicações clínicas práticas

Os achados deste estudo apresentam implicações relevantes para a abordagem clínica de pacientes com IT grave. Nesta coorte, o TRI-SCORE demonstrou desempenho prognóstico consistente, podendo contribuir para a estratificação de risco e para a tomada de decisão terapêutica no contexto do *heart team*. Até onde se tem conhecimento, este é o primeiro estudo nacional a avaliar sistematicamente esse escore em uma ampla população com IT grave, fornecendo evidência local e parâmetros iniciais para sua calibração e aplicação em pacientes brasileiros.

Embora a natureza observacional do estudo imponha cautela na adoção ampla do TRI-SCORE, os resultados obtidos geram hipóteses que podem, futuramente, orientar protocolos de seguimento e critérios de seleção para intervenção. Nesse sentido, a estratificação precoce pelo TRI-SCORE, associada à reavaliação periódica de sua trajetória, pode ser útil na prática clínica. Pacientes inicialmente classificados como de baixo risco podem se beneficiar de vigilância mais estreita, considerando a possibilidade de intervenção em fases mais precoces da doença, estratégia alinhada a achados de registros multicêntricos que estratificam desfechos com base nesse escore.^20^

A associação entre marcadores laboratoriais (p.ex., hematócrito, leucócitos, plaquetas e creatinina) e mortalidade, bem como entre sinais ecocardiográficos de congestão direita (dilatação da VCI com redução do colabamento inspiratório) e derrame pericárdico, reforça a importância do monitoramento longitudinal desses parâmetros. Na prática, isso se traduz na necessidade de atenção sistemática a sinais de disfunção orgânica, particularmente renal, hematológica e hepática, que podem preceder desfechos adversos e servir como marcadores objetivos para intensificação terapêutica e discussão precoce no *heart team*.

A associação independente entre o uso de rivaroxabana e menor mortalidade sugere que, além das indicações formais (p.ex., FA), podem existir efeitos diferenciais relacionados à maior previsibilidade farmacocinética e à menor variabilidade da RNI em contextos de congestão hepática. No entanto, esse achado não permite inferência causal e deve ser interpretado com cautela, especialmente diante da possibilidade de viés de canalização terapêutica.

### Limitações do estudo

O delineamento retrospectivo e unicêntrico expõe o estudo a potenciais vieses de seleção e às limitações inerentes ao uso de dados secundários, incluindo inconsistências no preenchimento de prontuários e ausência de seguimento ativo dos pacientes. Do ponto de vista metodológico, a extração automatizada de dados por meio de PLN apresentou limitações, como lacunas em determinadas variáveis para parte dos indivíduos analisados (Tabela C, Material Suplementar II), o que exigiu complementação ativa manual.

Além disso, o desenho adotado foi transversal, baseado no ecocardiograma mais recente disponível para análise das variáveis, não contemplando avaliação longitudinal da evolução ecocardiográfica dos pacientes.

A aferição do desfecho mortalidade restringiu-se ao âmbito institucional, o que pode ter levado à subestimação de eventos em razão da ausência de registros externos. A indisponibilidade do tempo até o óbito impossibilitou a realização de análises de sobrevida, sendo adotada modelagem multivariável com desfecho binário em janela fixa. Ademais, não foi possível distinguir de forma sistemática entre óbitos perioperatórios e tardios, tampouco identificar perdas de seguimento, devido à ausência de monitoramento longitudinal.

Por fim, embora as estratégias terapêuticas sejam clinicamente relevantes no contexto da IT grave, especialmente em pacientes com etiologia secundária e história de intervenção prévia em valvas esquerdas, o delineamento do estudo não permitiu a comparação de desfechos conforme a abordagem terapêutica adotada.

### Perspectivas futuras

Os achados deste estudo reforçam a necessidade de ampliação da produção de dados sobre IT no Brasil, particularmente por meio de estudos multicêntricos que possibilitem a validação externa do TRI-SCORE e a avaliação comparativa com outros modelos prognósticos, como a *Isolated Tricuspid Valve Surgery Risk Calculator* da STS.

Estudos prospectivos serão fundamentais para elucidar o papel de intervenções farmacológicas, especialmente da anticoagulação, nos desfechos clínicos de pacientes com IT grave.

À luz dos avanços recentes nas terapias transcateter, a caracterização do perfil de risco da população brasileira torna-se essencial para otimizar a seleção de candidatos e maximizar os benefícios terapêuticos. Nesse contexto, a identificação precoce de marcadores laboratoriais e ecocardiográficos associados a pior prognóstico pode favorecer a indicação de intervenção em fases menos avançadas da doença, reduzindo o impacto do encaminhamento tardio, historicamente associado a piores resultados.

## Conclusões

Este estudo descreve uma ampla coorte de pacientes com IT grave identificados por meio de técnicas de IA aplicadas a laudos ecocardiográficos. A abordagem automatizada demonstrou-se viável e acurada, permitindo a caracterização abrangente de aspectos clínicos, laboratoriais e ecocardiográficos de uma população predominantemente composta por mulheres idosas, com etiologia secundária, elevada carga de comorbidades, especialmente hipertensão arterial sistêmica e FA, e frequente associação com valvopatia mitral significativa.

A estratificação de risco evidenciou elevada proporção de pacientes com alto risco cirúrgico, sendo o TRI-SCORE um preditor independente de mortalidade, o que reforça o impacto prognóstico da IT grave e a complexidade de seu manejo.

Em conjunto, esses achados destacam o potencial da IA na construção de grandes coortes, no suporte a modelos preditivos e na otimização de estratégias de estratificação de risco, podendo contribuir para a definição mais precisa do momento ideal para intervenção valvar (Figura Central).

## Data Availability

Os conteúdos subjacentes ao texto da pesquisa estão contidos no manuscrito.

## *Material suplementar

Para Material Suplementar I, por favor, clique aqui.

Para Material Suplementar II, por favor, clique aqui.
