# Supplementary material for: Aplicação da Inteligência Artificial para Detecção, Caracterização e Estratificação de Risco de Pacientes com Insuficiência Tricúspide Grave
Source: Arq Bras Cardiol. 2026 May 29;123(4):e20250743. [Article in Portuguese] doi: 10.36660/abc.20250743 (PMC13398803; doi:10.36660/abc.20250743)
Supplement: Supplementary file 1 [file 0066-782x-abc-123-4-e20250743-Supp01.pdf]

MATERIAL SUPLEMENTAR I

- **Tabela A.** Variáveis de interesse na extração de dados

DEMOGRÁFICAS	ANTROPOMÉTRICAS	CLÍNICAS	LABORATORIAIS	ECOCARDIOGRÁFICAS	ESCORES DE RISCO
Idade	Peso	Tabagismo	Hematócritos	Provável etiologia da IT grave	EuroSCORE II
Sexo	Altura	Etilismo	Leucócitos	Medida da aorta (seios Valsalva)	TRI-SCORE
	IMC	HAS	Plaquetas	Medida da aorta ascendente (unidade: mm)	
		DM	Creatinina sérica	Diâmetro do átrio esquerdo (unidade: mm)	
		DLP	Taxa de filtração glomerular	Volume indexado do átrio esquerdo (unidade: mL/m²)	
		DRC	NT-pró-BNP	Diâmetro diastólico final do VE (unidade: mm)	
		DAOP	Bilirrubinas elevadas	Diâmetro sistólico final do VE (unidade: mm)	
		DAC		Espessura diastólica do septo interventricular (unidade: mm)	
		FA		Espessura diastólica da parede posterior (unidade: mm)	
		Doença pulmonar		Massa ventricular esquerda (unidade: g)	
		Hipertensão pulmonar		Massa ventricular esquerda indexada (unidade: g/m²)	
		Estágio NYHA		Espessura relativa de parede	
		Edema		Fração de ejeção do ventrículo esquerdo (unidade: porcentagem)	
		Turgência jugular		Contratilidade do ventrículo esquerdo (análise visual)	
		DCEI		Função sistólica do ventrículo direito (análise visual)	
		IECA		Pressão sistólica da artéria pulmonar estimada pelo refluxo tricuspídeo (unidade: mmHg)	
		BRA		Diâmetro da via de saída do VE (unidade: mm)	
		Insulina		Diâmetro da veia cava inferior (unidade: mm)	
		Furosemida		Variação respiratória da veia cava inferior (análise visual)	
		Furosemida dose > 125mg/dia		Pericárdio (análise visual quanto à presença de derrame)	
		Anticoagulante		Avaliação valvar mitral	

	Cirurgia cardíaca prévia	Avaliação valvar mitral
		Avaliação valvar tricúspide

- **Tabela B.** Descritivo da amostra total (óbito *x* não óbito)

**Variável**	**Categoria / Medidas**	**Total** N (%) / Medidas	**Óbito** **Não** N (%) / Medidas	**Sim**	**p-valor**
Gênero	Feminino	515 (64,1)	425 (63,9)	90 (65,2)	0,771
	Masculino	288 (35,9)	240 (36,1)	48 (34,8)	
Idade (anos)	N	803	665	138	0,362
	Variação	18 – 107	30 – 99	18 – 107	
	Média ± DP	68,8 ± 13,5	68,6 ± 13,3	69,7 ± 14,2	
IMC	N	803	665	138	0,024
	Variação	13,9 – 71,8	13,9 – 71,8	16,9 – 42,4	
	Média ± DP	26,7 ± 5,4	26,8 ± 5,4	25,9 ± 5,4	
Tabagismo	Não	512 (63,8)	426 (64,1)	86 (62,3)	0,084
	Ex-tabagista	251 (31,3)	211 (31,7)	40 (29,0)	
	Sim	40 (5,0)	28 (4,2)	12 (8,7)	
Etilismo	Não	688 (85,7)	562 (84,5)	126 (91,3)	0,038
	Sim	115 (14,3)	103 (15,5)	12 (8,7)	

Os valores são apresentados como n (%) e como média ± desvio padrão ou mediana (intervalo interquartil)†, de acordo com a distribuição dos dados.

Variável	Medidas / Categoria	Total	Óbito		p-valor
			Não	Sim	
		N (%) / Medidas	N (%) / Medidas		
EuroSCORE II	N	803	665	138	<0,001
	Variação	1,6 – 58,0	1,6 – 58,0	1,7 – 51,7	
	Mediana†	13,4 (8,1–21,3)	12,5 (7,5–19,5)	19,0 (12,5–27,2)	
EuroSCORE II	Baixo	43 (5,4)	39 (5,9)	4 (2,9)	<0,001
Risco	Intermediário	155 (19,3)	143 (21,5)	12 (8,7)	
	Alto	605 (75,3)	483 (72,6)	122 (88,4)	
EuroSCORE II	Baixo +				
Risco	Intermediário	198 (24,7)	182 (27,4)	16 (11,6)	<0,001
	Alto	605 (75,3)	483 (72,6)	122 (88,4)	

Os valores são apresentados como n (%) e como média ± desvio padrão ou mediana (intervalo interquartil)†, de acordo com a distribuição dos dados.

Faixa escore: considerando:
(<4%) = Baixo risco; (4% - 8%) = Risco Intermediário; (> 8%) = Alto risco

Variável	Medidas / Categoria	Total	Óbito		p-valor
			Não	Sim	
		N (%) / Medidas	N (%) / Medidas		
TRI-SCORE	N	803	665	138	<0,001
simples	Variação	2 – 11	2 – 11	4 – 11	
	Mediana†	7 (6–8)	6 (5–7)	7 (6–9)	
TRI-SCORE	Baixo	20 (2,5)	20 (3,0)	0	NA
Simples	Intermediário	180 (22,4)	164 (24,7)	16 (11,6)	
Risco	Alto	603 (75,1)	481 (72,3)	122 (88,4)	

TRI-SCORE	Baixo +				
Simples	Intermediário	200 (24,9)	184 (27,7)	16 (11,6)	<0,001
Risco	Alto	603 (75,1)	481 (72,3)	122 (88,4)	
TRI-SCORE	N	803	665	138	<0,001
Odds padronizado	Variação	1 – 14	1 – 14	3 – 14	
	Mediana†	7 (6–9)	7 (5–8)	9 (7–10)	
TRI-SCORE					
Odds padronizado Risco	Baixo	51 (6,3)	50 (7,5)	1 (0,7)	<0,001
	Intermediário	145 (18,1)	130 (19,6)	15 (10,9)	
	Alto	607 (75,6)	485 (72,9)	122 (88,4)	
TRI-SCORE	Baixo +				
Odds padronizado Risco	Intermediário	196 (24,4)	180 (27,1)	16 (11,6)	<0,001
	Alto	607 (75,6)	485 (72,9)	122 (88,4)	

Os valores são apresentados como n (%) e como média ± desvio padrão ou mediana (intervalo interquartil)†, de acordo com a distribuição dos dados.

Faixa TRI-SCORE, considerando:
(≤3) = Baixo risco; (4 - 5) = Risco Intermediário; (≥ 6) = Alto risco

			Óbito		
**Variável**	**Categoria**	**Total**	**Não**	**Sim**	**p-valor**
		**N (%)**	**N (%)**		
AVC	Não	685 (85,3)	573 (86,2)	112 (81,2)	0,131
	Sim	118 (14,7)	92 (13,8)	26 (18,8)	
HAS	Não	227 (28,3)	199 (29,9)	28 (20,3)	0,022
	Sim	576 (71,7)	466 (70,1)	110 (79,7)	
DM	DMID	42 (5,2)	32 (4,8)	10 (7,2)	0,243

	Não	562 (70,0)	473 (71,1)	89 (64,5)	
	DMNID	199 (24,8)	160 (24,1)	39 (28,3)	
DLP	Não	382 (47,6)	320 (48,1)	62 (44,9)	0,494
	Sim	421 (52,4)	345 (51,9)	76 (55,1)	
DRC	Não	596 (74,2)	503 (75,6)	93 (67,4)	0,044
	Sim	207 (25,8)	162 (24,4)	45 (32,6)	
DAOP	Não	756 (94,2)	628 (94,4)	128 (92,8)	0,444
	Sim	47 (5,8)	37 (5,6)	10 (7,2)	
DAC	Não	634 (79,0)	534 (80,3)	100 (72,5)	0,040
	Sim	169 (21,0)	131 (19,7)	38 (27,5)	
FA	Não	294 (36,6)	232 (34,9)	62 (44,9)	0,026
	Sim	509 (69,4)	433 (65,1)	76 (55,1)	
Doença pulmonar	Não	671 (83,6)	559 (84,1)	112 (81,2)	0,403
	Sim	132 (16,4)	106 (15,9)	26 (18,8)	
Hipertensão pulmonar moderada (n=774)	Não	371 (47,9)	290 (45,4)	81 (59,6)	0,003
	Sim	403 (52,1)	348 (54,6)	55 (40,4)	
Hipertensão pulmonar grave n=774	Não	440 (56,8)	374 (58,6)	66 (48,5)	0,031
	Sim	334 (43,2)	264 (41,4)	70 (51,5)	
Hipertensão pulmonar	Não	37 (4,8)	26 (4,1)	11 (8,1)	0,005
n=774	Moderada	403 (52,1)	348 (54,5)	55 (40,4)	
	Grave	334 (43,1)	264 (41,4)	70 (51,5)	

Os valores são apresentados como n (%) e como média ± desvio padrão ou mediana (intervalo interquartil)†, de acordo com a distribuição dos dados.

Variável	Categoria / Medidas	Total<br>N (%) / Medidas	Óbito<br>Não<br>N (%) / Medidas	Óbito<br>Sim	p-valor
NYHA	I	247 (30,8)	222 (33,4)	25 (18,4)	<0,001
n=801	II	427 (53,3)	345 (51,9)	82 (60,3)	
	III	72 (9,0)	61 (9,2)	11 (8,1)	
	IV	55 (6,9)	37 (5,6)	18 (13,2)	
NYHA	I + II	674 (84,1)	567 (85,3)	107 (78,7)	0,055
	III + IV	127 (15,9)	98 (14,7)	29 (21,3)	

Os valores são apresentados como n (%) e como média ± desvio padrão ou mediana (intervalo interquartil)†, de acordo com a distribuição dos dados.

Variável	Categoria / Medidas	Total<br>N (%) / Medidas	Óbito<br>Não<br>N (%) / Medidas	Óbito<br>Sim	p-valor
Dispositivo cardíaco eletrônico implantável	Não	680 (84,7)	563 (84,7)	117 (84,8)	0,971
	Sim	123 (15,3)	102 (15,3)	21 (15,2)	
Etiologia da IT	Primária	26 (3,2)	22 (3,3)	4 (2,9)	0,999
	Secundária	777 (96,8)	643 (96,7)	134 (97,1)	
Edema	Não	741 (92,3)	608 (91,4)	133 (96,4)	0,048
	Sim	62 (7,7)	57 (8,6)	5 (3,6)	
Turgência jugular	Não	803 (100,0)	665 (100,0)	138 (100,0)	NA
	Sim	0	0	0	
IECA	Não	532 (66,2)	439 (66,0)	93 (67,4)	0,756
	Sim	271 (33,8)	226 (34,0)	45 (32,6)	

BRA	Não	529 (65,9)	425 (63,9)	104 (75,4)	0,010
	Sim	274 (34,1)	240 (36,1)	34 (24,6)	
Insulina	Não	744 (92,7)	621 (93,4)	123 (89,1)	0,081
	Sim	59 (7,3)	44 (6,6)	15 (10,9)	
Varfarina	Não	535 (66,6)	436 (65,6)	99 (71,7)	0,162
	Sim	268 (33,4)	229 (34,4)	39 (28,3)	
Rivaroxabana	Não	559 (69,6)	456 (68,6)	103 (74,6)	0,159
	Sim	244 (30,4)	209 (31,4)	35 (25,4)	
Anticoagulante	Não	291 (36,2)	227 (34,1)	64 (46,4)	0,025
	Varfarina	268 (33,4)	229 (34,4)	39 (28,3)	
	Rivaroxabana	244 (30,4)	209 (31,4)	35 (25,4)	
Furosemida	Não	193 (24,0)	166 (25,0)	27 (19,6)	0,177
	Sim	610 (76,0)	499 (75,0)	111 (80,4)	
Furosemida acima de 125 mg/dia	Não	734 (91,4)	617 (92,8)	117 (84,8)	0,002
	Sim	69 (8,6)	48 (7,2)	21 (15,2)	
Hematócritos	N	792	654	138	<0,001
	Variação	11,8 – 97,8	11,8 – 97,8	14,6 – 51,5	
	Mediana†	37,5 (33,1–41,6)	38,2 (34,6–42,0)	31,2 (24,7–36,5)	
Leucócitos	N	793	655	138	<0,001
	Variação	1,70 – 58,92	1,83 – 58,92	1,70 – 26,90	
	Mediana†	6,8 (5,4–8,7)	6,5 (5,3–8,3)	9,2 (6,9–14,1)	

Os valores são apresentados como n (%) e como média ± desvio padrão ou mediana (intervalo interquartil)†, de acordo com a distribuição dos dados.
NA= não avaliável estatisticamente

Variável	Categoria / Medidas	Total	Óbito		p-valor
			Não	Sim	
		N (%) / Medidas	N (%) / Medidas		
Plaquetas	N	793	655	138	<0,001
	Variação	23000 – 755000	23000 – 755000	23000 – 492000	
	Mediana†	189000 (147000–236000)	194000 (155000–241000)	157000 (92000–203000)	
Creatinina	N	793	655	138	<0,001
	Variação	0,5 – 12,2	0,5 – 12,2	0,6 – 8,1	
	Mediana†	1,3 (1,0–1,7)	1,2 (0,9–1,5)	1,9 (1,3–2,8)	
Taxa de filtração glomerular	N	793	655	138	<0,001
	Variação	5 – 96	5 – 96	6 – 94	
	Mediana†	52 (35–72)	55 (40–75)	31 (18–45)	
Bilirrubinas totais aumentadas n=782	Não	647 (82,7)	566 (87,9)	81 (58,7)	<0,001
	Sim	135 (17,3)	78 (12,1)	57 (41,3)	
Pró peptídeo Natriurético tipo B	N	773	640	133	<0,001
	Variação	36 – 166982	36 – 166982	115 – 154000	
	Mediana†	2517 (905–9060)	1878 (771–6123)	12400 (5720–32000)	
Atual gravidade do refluxo tricúspide	Mínimo	11 (1,4)	11 (1,6)	0	NA
	Discreto	47 (5,9)	43 (6,5)	4 (2,9)	
	Discreto a moderado	25 (3,1)	22 (3,3)	3 (2,2)	
	Moderado	50 (6,2)	45 (6,8)	5 (3,6)	
	IT grave	670 (83,4)	544 (81,8)	126 (91,3)	

Fez cirurgia em outra valva nesse período n=133	Não	78 (58,6)	70 (57,9)	8 (66,7)	NA
	Sim	50 (37,6)	46 (38,0)	4 (33,3)	
	Transplante cardíaco	5 (3,8)	5 (4,1	0	
Anel tricúspide (mm)	N	155	139	16	0,395
	Variação	28 – 67	28 – 58	30 – 67	
	Mediana†	41 (39–44)	41 (39–44)	43 (40–44)	
Raiz da aorta (Seios de Valsalva) (mm)	N	798	662	136	0,014
	Variação	23 – 53	23 – 53	26 – 45	
	Mediana†	33 (31–36)	33 (31–36)	32 (30–35)	

Os valores são apresentados como n (%) e como média ± desvio padrão ou mediana (intervalo interquartil)†, de acordo com a distribuição dos dados.

			Óbito		
**Variável**	**Medidas**	**Total**	**Não**	**Sim**	**p-valor**
		**Medidas**	**Medidas**		
Aorta ascendente proximal (mm)	N	703	588	115	0,098
	Variação	23 – 68	23 – 68	25 – 59	
	Mediana†	34 (31–37)	34 (31–37)	33 (30–36)	
Átrio esquerdo - Diâmetro (mm)	N	801	663	138	0,361
	Variação	25 – 120	25 – 120	28 – 118	
	Mediana†	51 (46–58)	51 (46–58)	51 (48–58)	
Átrio Esquerdo - volume indexado (ml/m²)	N	790	655	135	0,419
	Variação	23 – 1048	23 – 1048	31 – 550	

	Mediana†	67 (54–87)	67 (53–87)	68 (57–87)	
Diâmetro diastólico final do VE (mm)	N	796	658	138	0,652
	Variação	29 – 99	32 – 99	29 – 85	
	Mediana†	54 (48–60)	54 (48–60)	54 (46–62)	
Diâmetro sistólico final do VE (mm)	N	611	506	105	0,201
	Variação	21 – 80	21 – 79	22 – 80	
	Mediana†	38 (31–48)	38 (32–47)	40 (31–53)	
Espessura diastólica do septo (mm)	N	793	655	138	0,962
	Variação	6 – 30	6 – 30	6 – 17	
	Mediana†	9 (8–10)	9 (8–10)	9 (8–10)	
Espessura diastólica da parede posterior (mm)	N	799	661	138	0,759
	Variação	1 – 18	1 – 18	6 – 16	
	Mediana†	9 (8–10)	9 (8–10)	9 (8–10)	
Massa ventricular esquerda (g)	N	801	663	138	0,768
	Variação	56,1– 458,7	68,9 – 458,7	56,1 – 428,7	
	Mediana†	189,3 (150,1–234,3)	188,0 (151,7–236,3)	193,5 (147,9–231,0)	
Massa do VE indexada (g/m²)	N	801	663	138	0,896
	Variação	33 – 265	42 – 265	33 – 248	
	Mediana†	110 (90–134)	110 (90–134)	110 (88–136)	

Espessura relativa da parede posterior	N	801	663	138	0,670
	Variação	0,1 – 0,9	0,1 – 0,9	0,2 – 0,8	
	Mediana†	0,3 (0,3–0,4)	0,3 (0,3–0,4)	0,3 (0,3–0,4)	
Fração de ejeção VE (%)	N	803	665	138	0,011
	Variação	12 – 70	12 – 70	15 – 68	
	Mediana†	48 (31–58)	48 (32–59)	42 (25–56)	

Os valores são apresentados como n (%) e como média ± desvio padrão ou mediana (intervalo interquartil)†, de acordo com a distribuição dos dados.
NA= não avaliável estatisticamente

Variável	Categoria / Medidas	Total	Óbito		p-valor
			Não	Sim	
		N (%)/Medidas	N (%) / Medidas		
Função sistólica do VE	preservada	336 (41,8)	288 (43,3)	48 (34,8)	0,051
	disfunção discreta	143 (17,8)	117 (17,6)	26 (18,8)	
	disfunção moderada	136 (16,9)	116 (17,4)	20 (14,5)	
	disfunção importante	188 (23,4)	144 (21,7)	44 (31,9)	
Contratilidade preservada	Não	425 (52,9)	344 (51,7)	81 (58,7)	0,136
	Sim	378 (47,1)	321 (48,3)	57 (41,3)	
PSAP (mmHg)	N	771	637	134	0,001
	Variação	21 – 195	21 – 195	22 – 125	
	Mediana†	53 (45–65)	52 (43–64)	59 (47–70)	
Função sistólica do VD	Função preservada	367 (45,7)	333 (50,1)	34 (24,6)	<0,001
	Disfunção	436 (54,3)	332 (49,9)	104 (75,4)	
VSVE (mm)	N	155	129	26	0,810
	Variação	17 – 29	17 – 29	17 – 28	
	Mediana†	21 (20–22)	21 (20–22)	21 (20–22)	
Veia cava inferior (mm)	N	759	630	129	<0,001
	Variação	6 – 91	6 – 91	15 – 37	
	Mediana†	23 (20–27)	23 (20–26)	25 (23–28)	
Colabamento	Menor que 50%	536 (67,6)	426 (64,7)	110 (81,5)	<0,001
n=793	Maior que 50%	257 (32,4)	232 (35,3)	25 (18,5)	

Derrame pericárdico presente	Não	660 (82,2)	561 (84,4)	99 (71,7)	<0,001
	Sim	143 (17,8)	104 (15,6)	39 (28,3)	
Valvopatia mitral associada	Não	253 (31,5)	210 (31,6)	43 (31,2)	0,923
	Sim	550 (68,5)	455 (68,4)	95 (68,8)	
Valvopatia aórtica associada	Não	620 (77,2)	515 (77,4)	105 (76,1)	0,730
	Sim	183 (22,8)	150 (22,6)	33 (23,9)	
Prótese mitral biológica	Não	694 (86,4)	574 (86,3)	120 (87,0)	0,841
	Sim	109 (13,6)	91 (13,7)	18 (13,0)	
Prótese mitral metálica	Não	738 (91,9)	607 (91,3)	131 (94,9)	0,153
	Sim	65 (8,1)	58 (8,7)	7 (5,1)	
Prótese aórtica biológica	Não	744 (92,6)	612 (92,0)	132 (95,6)	0,138
	Sim	59 (7,4)	53 (8,0)	6 (4,4)	
Prótese aórtica metálica	Não	756 (94,2)	624 (93,8)	132 (95,6)	0,408
	Sim	47 (5,8)	41 (6,2)	6 (4,4)	
Plastia tricúspide	Não	769 (95,8)	633 (95,2)	136 (98,6)	0,074
	Sim	34 (4,2)	32 (4,8)	2 (1,4)	

Os valores são apresentados como n (%) e como média ± desvio padrão ou mediana (intervalo interquartil)†, de acordo com a distribuição dos dados.
NA= Não avaliável estatisticamente

Variável	Categoria/ Medidas	Total N (%) / Medidas	Óbito Não N (%) / Medidas	Óbito Sim N (%) / Medidas	p-valor
Mitral fina	Não	608 (75,7)	503 (75,6)	105 (76,1)	0,911
	Sim	195 (24,3)	162 (24,4)	33 (23,9)	
Mitral espessada	Não	397 (49,4)	337 (50,7)	60 (43,5)	0,124
	Sim	406 (50,6)	328 (49,3)	78 (56,5)	
Mitral calcificada	Não	680 (84,7)	560 (84,2)	120 (87,0)	0,415
	Sim	123 (15,3)	105 (15,8)	18 (13,0)	
Prótese mitral fina	Não	802 (99,9)	664 (99,8)	138 (100,0)	NA
	Sim	1 (0,1)	1 (0,2)	0	
Prótese mitral espessada	Não	803 (100,0)	665 (100,0)	138 (100,0)	NA
	Sim	0	0	0	
Prótese mitral calcificada	Não	803 (100,0)	665 (100,0)	138 (100,0)	NA
	Sim	0	0	0	
Gradiente diastólico máximo mitral (mmHg)	N	277	233	44	0,001
	Variação	4 – 43	4 – 43	6 – 40	
	Mediana†	14 (11–19)	14 (11–17)	19 (12–22)	
Gradiente diastólico médio mitral (mmHg)	N	275	231	44	<0,001
	Variação	2 – 26	2 – 19	2 – 26	
	Mediana†	5 (4–8)	5 (4–7)	9 (5–13)	
Refluxo mitral moderado	Não	757 (94,3)	627 (94,3)	130 (94,2)	0,970
	Sim	46 (5,7)	38 (5,7)	8 (5,8)	

Refluxo mitral importante	Não	744 (92,6)	617 (92,8)	127 (92,0)	0,758
	Sim	59 (7,4)	48 (7,2)	11 (8,0)	
Refluxo mitral protético	Não	803 (100,0)	665 (100,0)	138 (100,0)	NA
	Sim	0	0	0	
Aórtica fina	Não	738 (91,9)	606 (91,1)	132 (95,6)	0,076
	Sim	65 (8,1)	59 (8,9)	6 (4,4)	
Aórtica espessada	Não	186 (23,2)	166 (25,0)	20 (14,5)	0,008
	Sim	617 (76,8)	499 (75,0)	118 (85,5)	
Aórtica calcificada	Não	652 (81,2)	541 (81,4)	111 (80,4)	0,802
	Sim	151 (18,8)	124 (18,6)	27 (19,6)	

			**Óbito**		
**Variável**	**Categoria / Medidas**	**Total N (%)/Medidas**	**Não N (%) / Medidas**	**Sim**	**p-valor**
Prótese aórtica fina	Não	735 (91,5)	598 (89,9)	137 (99,3)	<0,001
	Sim	68 (8,5)	67 (10,1)	1 (0,7)	
Prótese aórtica espessada	Não	801 (99,8)	663 (99,7)	138 (100,0)	0,999
	Sim	2 (0,2)	2 (0,3)	0	
Prótese aórtica calcificada	Não	788 (98,1)	654 (98,3)	134 (97,1)	0,306
	Sim	15 (1,9)	11 (1,7)	4 (2,9)	
Gradiente sistólico máximo aórtico (mmHg)	N	252	212	40	0,066
	Variação	7 – 118	7 – 118	10 – 109	
	Mediana†	18 (13–31)	18 (13–30)	22 (15–43)	

Gradiente sistólico médio aórtico (mmHg)	N	170	142	28	0,003
	Variação	2 – 80	2 – 80	8 – 66	
	Mediana†	14 (9–23)	13 (9–22)	21 (11–31)	
Refluxo aórtico moderado	Não	777 (96,8)	642 (96,5)	135 (97,8)	0,438
	Sim	26 (3,2)	23 (3,5)	3 (2,2)	
Refluxo aórtico importante	Não	801 (99,8)	664 (99,8)	137 (99,3)	0,314
	Sim	2 (0,2)	1 (0,2)	1 (0,7)	
Refluxo aórtico protético	Não	803 (100,0	665 (100,0)	138 (100,0)	NA
	Sim	(0)	0	0	
Tricúspide fina	Não	140 (17,4)	125 (18,8)	15 (10,9)	0,026
	Sim	663 (82,6)	540 (81,2)	123 (89,1)	
Tricúspide espessada	Não	722 (89,9)	596 (89,6)	126 (91,3)	0,551
	Sim	81 (10,1)	69 (10,4)	12 (8,7)	
Tricúspide calcificada	Não	803 (100,0)	665 (100,0)	138 (100,0)	NA
	Sim		0	0	
Mobilidade tricúspide preservada	Não	185 (23,0)	152 (22,9)	33 (23,9)	0,789
	Sim	618 (77,0)	513 (77,1)	105 (76,1)	
Cirurgia cardíaca prévia	Sim	235 (29,3)	203 (30,5)	32 (23,2)	0,085
	Não	568 (70,7)	462 (69,5)	106 (76,8)	

Os valores são apresentados como n (%) e como média ± desvio padrão ou mediana (intervalo interquartil)†, de acordo com a distribuição dos dados.

NA= Não avaliável estatisticamente

- **Tabela C.** Análise univariada

Variável	Categoria	Óbito Não	Óbito Sim	RR	(95% IC)	p-valor
		N (%)	N (%)			
IMC	Normalidade	9 (1,3)	7 (5,1)	1,0	Ref.	
	Abaixo do peso	274 (41,2)	65 (47,1)	3,28	1,18 – 9,13	0,023
	Sobrepeso	230 (34,6)	38 (27,5)	0,70	0,45 – 1,08	0,105
	Obesidade	152 (22,9)	28 (20,3)	0,78	0,48 – 1,26	0,307
Tabagismo	Não	426 (64,1)	86 (62,3)	1,0	Ref.	
	Ex-tabagista	211 (31,7)	40 (29,0)	1,06	0,71 – 1,60	0,764
	Sim	28 (4,2)	12 (8,7)	2,26	1,06 – 4,81	0,034
Etilismo	Não	562 (84,5)	126 (91,3)	1,0	Ref.	
	Sim	103 (15,5)	12 (8,7)	0,52	0,28 – 0,97	0,041
EuroSCORE II Risco	Baixo	39 (5,9)	4 (2,9)	1,0	Ref.	
	Intermediário	143 (21,5)	12 (8,7)	0,82	0,25 - 2,68	0,740
	Alto	483 (72,6)	122 (88,4)	2,46	0,86 – 7,02	0,092
EuroSCORE II Risco	Baixo + Intermediário	182 (27,4)	16 (11,6)	1,0	Ref.	
	Alto	483 (72,6)	122 (88,4)	2,87	1,66 – 4,97	<0,001
TRI-SCORE Simples Risco	Baixo + Intermediário	184 (27,7)	16 (11,6)	1,0	Ref.	
	Alto	481 (72,3)	122 (88,4)	2,92	1,69 – 5,05	<0,001
TRI-SCORE Odds padronizado Risco	Baixo	50 (7,5)	1 (0,7)	1,0	Ref.	
	Intermediário	130 (19,6)	15 (10,9)	5,77	0,74 – 44,83	0,094
	Alto	485 (72,9)	122 (88,4)	12,58	1,72 – 91,95	0,013

TRI-SCORE						
Odds padronizado Risco	Baixo + Intermediário	180 (27,1)	16 (11,6)	1,0	Ref.	
	Alto	485 (72,9)	122 (88,4)	2,83	1,63 – 4,90	<0,001

IMC
< 18,50 (abaixo do peso)
18,50 – 24,99 (normalidade)
25,00 – 29,99 (sobrepeso)
≥ 30,00 (obesidade)

**Variável**	**Categoria**	**Óbito** **Não**	**Sim**	**RR**	**(95% IC)**	**p-valor**
		**N (%)**				
AVC	Não	573 (86,2)	112 (81,2)	1,0	Ref.	
	Sim	92 (13,8)	26 (18,8)	1,44	0,89 – 2,34	0,132
HAS	Não	199 (29,9)	28 (20,3)	1,0	Ref.	
	Sim	466 (70,1)	110 (79,7)	1,68	1,07 – 2,62	0,023
DLP	Não	320 (48,1)	62 (44,9)	1,0	Ref.	
	Sim	345 (51,9)	76 (55,1)	1,14	0,79 – 1,64	0,494
DRC	Não	503 (75,6)	93 (67,4)	1,0	Ref.	
	Sim	162 (24,4)	45 (32,6)	1,50	1,01 – 2,23	0,045
DAC	Não	534 (80,3)	100 (72,5)	1,0	Ref.	
	Sim	131 (19,7)	38 (27,5)	1,55	1,02 – 2,36	0,041
IECA	Não	439 (66,0)	93 (67,4)	1,0	Ref.	
	Sim	226 (34,0)	45 (32,6)	0,94	0,64 – 1,39	0,756
BRA	Não	425 (63,9)	104 (75,4)	1,0	Ref.	

	Sim	240 (36,1)	34 (24,6)	0,58	0,38 – 0,88	0,010
Insulina	Não	621 (93,4)	123 (89,1)	1,0	Ref.	
	Sim	44 (6,6)	15 (10,9)	1,72	0,93 – 3,19	0,085
Anticoagulante	Não	227 (34,1)	64 (46,4)	1,0	Ref.	
	Varfarina	229 (34,4)	39 (28,3)	0,60	0,39 – 0,94	0,024
	Rivaroxabana	209 (31,4)	35 (25,4)	0,59	0,38 – 0,93	0,024
Hematócrito*	Normal	234 (35,8)	22 (15,9)	1,0	Ref.	
	Alterado	420 (64,2)	116 (84,1)	2,94	1,81 – 4,76	<0,001
Leucócitos*	Normal	537 (82,0)	75 (54,4)	1,0	Ref.	
	Alterado	118 (18,0)	63 (45,6)	3,82	2,59 – 5,64	<0,001
Plaquetas*	Normal	499 (76,2)	73 (52,9)	1,0	Ref.	
	Alterado	156 (23,8)	65 (47,1)	2,85	1,95 – 4,16	<0,001
Creatinina*	Normal	335 (51,2)	30 (21,7)	1,0	Ref.	
	Alterado	320 (48,8)	108 (78,3)	3,77	2,44 - 5,81	<0,001

*Normal e alterado (Vide *cut-off*)

Variável	Categoria	Óbito Não	Sim	RR	(95% IC)	p-valor
		N (%)				
Pró peptídeo Natriurético tipo B*	Normal	18 (2,8)	1 (0,8)	1,0	Ref.	
	Alterado	622 (97,2)	132 (99,2)	3,82	0,50 – 28,86	0,194
Atual gravidade do refluxo tricúspide	Minimo	11 (1,6)	0	1,0	Ref.	
	Discreto	43 (6,5)	4 (2,9)			
	Discreto a moderado	22 (3,3)	3 (2,2)			
	Moderado	45 (6,8)	5 (3,6)			
	IT grave	544 (81,8)	126 (91,3)			
Atual gravidade do refluxo tricúspide	Sem IT grave	121 (18,2)	12 (8,7)	1,0	Ref.	0,008
	IT grave	544 (81,8)	126 (91,3)	2,33	1,25 – 4,36	
Fez cirurgia na Tricúspide nesse período n=133	Não	96 (79,3)	10 (83,3)	1,0	Ref.	
	Sim	20 (16,5)	2 (16,7)			
	Transplante cardíaco	5 (4,1)	0			
Fez cirurgia em outra valva nesse período n=133	Não	70 (57,9)	8 (66,7)	1,0	Ref.	
	Sim	46 (38,0)	4 (33,3)			
	Transplante cardíaco	5 (4,1)	0			
Anel tricúspide (mm)*	Normal	64 (46,0)	4 (25,0)	1,0	Ref.	
	Alterado	75 (54,0)	12 (75,0)	2,56	0,79 – 8,33	0,118
Raiz da Aorta (Seios de Valsalva) (mm)*	Normal	561 (84,7)	120 (88,2)	1,0	Ref.	
	Alterado	101 (15,3)	16 (11,8)	0,74	0,42 – 1,30	0,296
Aorta Ascendente Proximal (mm)*	Normal	408 (69,4)	90 (78,3)	1,0	Ref.	

	Alterado	180 (30,6)	25 (21,7)	0,63	0,39 – 1,01	0,057
Átrio esquerdo - diâmetro*	Normal	33 (5,0)	9 (6,5)	1,0	Ref.	
	Alterado	630 (95,0)	129 (93,5)	0,75	0,35 – 1,61	0,460
Átrio esquerdo - volume indexado*	Normal	5 (0,8)	0	1,0	Ref.	
	Alterado	650 (99,2)	135 (100,0)			
Diâmetro diastólico final do VE (mm)*	Normal	329 (50,0)	64 (46,4)	1,0	Ref.	
	Alterado	329 (50,0)	74 (53,6)	1,16	0,80 – 1,67	0,439
Diâmetro sistólico final do VE (mm)*	Normal	243 (48,0)	39 (37,1)	1,0	Ref.	
	Alterado	263 (52,0)	66 (62,9)	1,56	1,01 – 2,41	0,043

*Normal e alterado (Vide *cut-off*)

		Óbito				
**Variável**	**Categoria**	**Não**	**Sim**	**RR**	**(95% IC)**	**p-valor**
		**N (%)**				
Espessura diastólica do septo (mm)*	Normal	443 (67,6)	87 (63,0)	1,0	Ref.	
	Alterado	212 (32,4)	51 (37,0)	1,22	0,83 – 1,79	0,298
Espessura diastólica da parede posterior (mm)*	Normal	489 (74,0)	97 (70,3)	1,0	Ref.	
	Alterado	172 (26,0)	41 (29,7)	1,20	0,80 – 1,80	0,373
Massa ventricular Esquerda (g)*	Normal	296 (44,6)	68 (49,3)	1,0	Ref.	
	Alterado	367 (55,4)	70 (50,7)	0,83	0,57 – 1,20	0,321
Massa do VE indexada (g/m²)*	Normal	257 (38,8)	58 (42,0)	1,0	Ref.	
	Alterado	406 (61,2)	80 (58,0)	0,87	0,60 – 1,27	0,475

Espessura relativa da parede posterior*	Normal	583 (87,9)	111 (80,4)	1,0	Ref.	
	Alterado	80 (12,1)	27 (19,6)	1,77	1,09 – 2,87	0,020
Função sistólica do VE	preservada	288 (43,3)	48 (34,8)	1,0	Ref.	
	disfunção discreta	117 (17,6)	26 (18,8)	1,33	0,79 – 2,25	0,281
	disfunção moderada	116 (17,4)	20 (14,5)	1,03	0,59 – 1,82	0,906
	disfunção importante	144 (21,7)	44 (31,9)	1,83	1,16 – 2,89	0,009
PSAP (mmHg)	≤ 35 (normal)	59 (9,3)	10 (7,5)	1,0	Ref.	
	36-40 (limítrofe)	71 (11,1)	9 (6,7)	0,75	0,28 – 1,96	0,555
	> 40 (alterado)	507 (79,6)	115 (85,8)	1,34	0,66 – 2,69	0,415
Contratilidade preservada	Não	344 (51,7)	81 (58,7)	1,0	Ref.	
	Sim	321 (48,3)	57 (41,3)	0,75	0,52 – 1,09	0,137
VSVE (mm)	Normal	104 (80,6)	21 (80,7)	1,0	Ref.	
	Alterado	25 (19,4)	5 (19,2)	0,99	0,34 – 2,88	0,986
Veia cava inferior (mm)	Normal	231 (36,7)	18 (14,0)	1,0	Ref.	
	Alterado	399 (63,3)	111 (86,0)	3,57	2,11 – 6,03	<0,001
Colabamento	Menor que 50%	426 (64,7)	110 (81,5)	1,0	Ref.	
N=793	Maior que 50%	232 (35,3)	25 (18,5)	0,42	0,26 – 0,66	<0,001
Derrame pericárdico presente	Não	561 (84,4)	99 (71,7)	1,0	Ref.	
	Sim	104 (15,6)	39 (28,3)	2,12	1,39 – 3,25	0,001

Variável	Categoria	Óbito<br>Não	 Sim	RR	(95% IC)	p-valor
		N (%)				
Valvopatia mitral associada	Não	210 (31,6)	43 (31,2)	1,0	Ref.	
	Sim	455 (68,4)	95 (68,8)	1,02	0,69 – 1,51	0,923
Valvopatia aórtica associada	Não	515 (77,4)	105 (76,1)	1,0	Ref.	
	Sim	150 (22,6)	33 (23,9)	1,08	0,70 – 1,66	0,730
Prótese mitral biológica	Não	574 (86,3)	120 (87,0)	1,0	Ref.	
	Sim	91 (13,7)	18 (13,0)	0,95	0,55 – 1,63	0,842
Prótese mitral metálica	Não	607 (91,3)	131 (94,9)	1,0	Ref.	
	Sim	58 (8,7)	7 (5,1)	0,56	0,25 – 1,25	0,158
Prótese aórtica biológica	Não	612 (92,0)	132 (95,6)	1,0	Ref.	
	Sim	53 (8,0)	6 (4,4)	0,52	0,22 – 1,25	0,144
Prótese aórtica metálica	Não	624 (93,8)	132 (95,6)	1,0	Ref.	
	Sim	41 (6,2)	6 (4,4)	0,69	0,29 – 1,66	0,410
Plastia tricúspide	Não	633 (95,2)	136 (98,6)	1,0	Ref.	
	Sim	32 (4,8)	2 (1,4)	0,29	0,07 – 1,23	0,093
Mitral fina	Não	503 (75,6)	105 (76,1)	1,0	Ref.	
	Sim	162 (24,4)	33 (23,9)	0,97	0,63 – 1,50	0,904
Mitral espessada	Não	337 (50,7)	60 (43,5)	1,0	Ref.	
	Sim	328 (49,3)	78 (56,5)	1,33	0,92 – 1,93	0,128
Mitral calcificada	Não	560 (84,2)	120 (87,0)	1,0	Ref.	
	Sim	105 (15,8)	18 (13,0)	0,80	0,47 – 1,37	0,412

Refluxo mitral moderado	Não	627 (94,3)	130 (94,2)	1,0	Ref.	
	Sim	38 (5,7)	8 (5,8)	1,04	0,47 – 2,29	0,920
Refluxo mitral importante	Não	617 (92,8)	127 (92,0)	1,0	Ref.	
	Sim	48 (7,2)	11 (8,0)	1,11	0,56 – 2,19	0,765
Aórtica fina	Não	606 (91,1)	132 (95,6)	1,0	Ref.	
	Sim	59 (8,9)	6 (4,4)	0,46	0,20 – 1,10	0,082
Aórtica espessada	Não	166 (25,0)	20 (14,5)	1,0	Ref.	
	Sim	499 (75,0)	118 (85,5)	1,95	1,18 – 3,24	0,009
Aórtica calcificada	Não	541 (81,4)	111 (80,4)	1,0	Ref.	
	Sim	124 (18,6)	27 (19,6)	1,06	0,66 – 1,68	0,814

*Normal e alterado (Vide *cut-off*)

		Óbito				
**Variável**	**Categoria**	**Não**	**Sim**	**RR**	**(95% IC)**	**p-valor**
		N (%)				
Prótese aórtica fina	Não	598 (89,9)	137 (99,3)	1,0	Ref.	
	Sim	67 (10,1)	1 (0,7)	0,07	0,01 – 0,48	0,007
Prótese aórtica espessada	Não	663 (99,7)	138 (100,0)			
	Sim	2 (0,3)	0			
Prótese aórtica calcificada	Não	654 (98,3)	134 (97,1)	1,0	Ref.	
	Sim	11 (1,7)	4 (2,9)	1,77	0,55 – 5,64	0,335
Refluxo aórtico	Não	642 (96,5)	135 (97,8)	1,0	Ref.	
moderado	Sim	23 (3,5)	3 (2,2)	0,62	0,18 – 2,09	0,439

Tricúspide fina	Não	125 (18,8)	15 (10,9)	1,0	Ref.	
	Sim	540 (81,2)	123 (89,1)	1,90	1,08 – 3,37	0,027
Tricúspide espessada	Não	596 (89,6)	126 (91,3)	1,0	Ref.	0,545
	Sim	69 (10,4)	12 (8,7)	0,82	0,43 – 1,56	
Mobilidade tricúspide preservada	Não	152 (22,9)	33 (23,9)	1,0	Ref.	0,802
	Sim	513 (77,1)	105 (76,1)	0,95	0,61 – 1,46	

		**Óbito**				
**Variável**	**Categoria**	**Não**	**Sim**	**RR**	**(95% IC)**	**p-valor**
		**N (%)**				
Gradiente Diastólico máximo mitral (mmHg)	< 5	1 (0,43)	0			
	≥ 5	232 (99,6)	44 (100,0)			
Gradiente Diastólico médio mitral (mmHg)	< 2	0	0			
	≥ 2	231 (100,0)	44 (100,0)			
Gradiente Sistólico máximo aórtico (mmHg)	< 20	114 (53,8)	18 (45,0)	1,0	Ref.	
	≥ 20	98 (46,2)	22 (55,0)	1,42	0,72 – 2,80	0,310
Gradiente Sistólico médio aórtico (mmHg)	< 10	45 (31,7)	5 (17,9)	1,0	Ref.	
	≥ 10	97 (68,3)	23 (82,1)	2,13	0,76 – 5,97	0,149

- **Tabela D.** Análise multivariada

		Modelo Univariado			Modelo Multivariado		
**Variável**	**Categoria**	**RR**	**(95% IC)**	**p-valor**	**RR**	**(95% IC)**	**p-valor**
TRI-SCORE Simples	Baixo + Intermediário	1,0	Ref.		1,0	Ref.	
Risco	Alto	2,92	1,69 – 5,05	<0,001	2,19	1,14 – 4,21	0,019
Anticoagulante	Não	1,0	Ref.		1,0	Ref.	
	Varfarina	0,60	0,39 – 0,94	0,024	0,62	0,37 – 1,04	0,072
	Rivaroxabana	0,59	0,38 – 0,93	0,024	0,44	0,26 – 0,76	0,003
Hematócritos*	Normal	1,0	Ref.		1,0	Ref.	
	Alterado	2,94	1,81 – 4,76	<0,001	2,30	1,32 – 4,01	0,003
Leucócitos*	Normal	1,0	Ref.		1,0	Ref.	
	Alterado	3,82	2,59 – 5,64	<0,001	3,08	1,96 – 4,83	<0,001
Plaquetas*	Normal	1,0	Ref.		1,0	Ref.	
	Alterado	2,85	1,95 – 4,16	<0,001	2,25	1,45 – 3,49	<0,001
Creatinina*	Normal	1,0	Ref.		1,0	Ref.	
	Alterado	3,77	2,44 - 5,81	<0,001	3,13	1,91 - 5,14	<0,001
Aórtica espessada	Não	1,0	Ref.		1,0	Ref.	
	Sim	3,57	2,11 – 6,03	<0,001	1,98	1,09 – 3,61	0,025
Derrame pericárdico presente	Não	1,0	Ref.		1,0	Ref.	
	Sim	2,12	1,39 – 3,25	0,001	1,71	1,03 – 2,82	0,038
Veia cava inferior* (mm)	Normal	1,0	Ref.		1,0	Ref.	
	Alterado	3,57	2,11 – 6,03	<0,001	2,44	1,37 – 4,35	0,002

*Normal e alterado (Vide *cut-off*)
