# Supplementary material for: Aplicação da Inteligência Artificial para Detecção, Caracterização e Estratificação de Risco de Pacientes com Insuficiência Tricúspide Grave
Source: Arq Bras Cardiol. 2026 May 29;123(4):e20250743. [Article in Portuguese] doi: 10.36660/abc.20250743 (PMC13398803; doi:10.36660/abc.20250743)
Supplement: Supplementary file 2 [file 0066-782x-abc-123-4-e20250743-Supp02.pdf]

MATERIAL SUPLEMENTAR II

- **Tabela A.** Pontos de corte (*cut-offs*) utilizados pela estatística

Variável	Cut-off
IMC (kg/m²)	< 18,50 (abaixo do peso)<br>18,50 – 24,99 (normalidade)<br>25,00 – 29,99 (sobrepeso)<br>30,00 – 34,99 (obesidade grau I)<br>35,00 – 39,99 (obesidade grau II)<br>≥ 40,00 (obesidade grau III)
PSAP (mmHg)	≤ 35 (normalidade)<br>36-40 (limítrofe)<br>> 40 (alterado)
Hematócritos (%)	<40,0 (alterado)<br>40,0 – 50,0 (normalidade)<br>>50,0 (alterado)
Leucócitos (10³/microlitro)	<4,5 (alterado)<br>4,5 – 11,0 (normalidade)<br>>11,0 (alterado)
Plaquetas (células/microlitro)	<150000 (alterado)<br>150000 – 400000 (normalidade)<br>>400000 (alterado)
Creatinina (mg/dl)	<0,50 (mulheres) - alterado<br>0,50 – 1,1 (mulheres) - normalidade<br>>1,1 (mulheres) – alterado

	<0,7 – (homens) - alterado<br>0,70 – 1,3 (homens) - normalidade<br>>1,3 – (homens) - alterado
Pró peptídeo Natriurético tipo B (pg/ml)	<125 (normalidade)<br>≥ 125 (alterado)
Anel tricúspide (diâmetro no Eco) (mm)	≤ 40 (normalidade)<br>> 40 (alterado)
Raiz da aorta (Seios de Valsalva) (mm)	<30 (homens) - alterado<br>30 – 40 (homens) - normalidade<br>>40 (homens) - alterado<br><br><27 (mulheres) - alterado<br>27 – 36 (mulheres) - normalidade<br>>36 (mulheres) - alterado
Aorta ascendente proximal (mm)	<26 (homens) - alterado<br>26 – 38 (homens) - normalidade<br>>38 (homens) – alterado<br><br><23 (mulheres) - alterado<br>23 – 35 (mulheres) - normalidade<br>>35 (mulheres) - alterado

Átrio esquerdo - Diâmetro (mm)	<30 (homens) - alterado<br>30 – 40 (homens) - normalidade<br>>40 (homens) - alterado<br><br><27 (mulheres) - alterado<br>27 – 38 (mulheres) - normalidade<br>> 38 (mulheres) - alterado
Átrio esquerdo - Volume Indexado (mL/m²)	<16 (alterado)<br>16 – 28 (normalidade)<br>>28 (alterado)
Diâmetro diastólico final do VE (mm)	<42 (homens) - alterado<br>42 – 58 (homens) - normalidade<br>>58 (homens) - alterado<br><br><38 (mulheres) - alterado<br>38 – 52 (mulheres) - normalidade<br>>52 (mulheres) - alterado
Diâmetro sistólico final do VE<br>(mm)	<25 (homens) - alterado<br>25 – 40 (homens) - normalidade<br>>40 (homens) - alterado<br><br><22 (mulheres) - alterado<br>22 – 35 (mulheres) - normalidade<br>>35 (mulheres) - alterado
Espessura diastólica do septo<br>(mm)	6 – 10 (homens) - normalidade<br>>10 - alterado<br><br>6 – 9 (mulheres) - normalidade<br>>9 – alterado

Espessura diastólica da parede posterior (mm)	<6 (homens) - alterado<br>6 – 10 (homens) - normalidade<br>>10 (homens) - alterado<br><br><6 (mulheres) - alterado<br>6 – 9 (mulheres) - normalidade<br>>9 (mulheres) - alterado
Massa ventricular esquerda (g)	<88 (homens) - alterado<br>88 – 224 (homens) - normalidade<br>>224 (homens) - alterado<br><br><67 (mulheres) - alterado<br>67 – 162 (mulheres) - normalidade<br>>162 (mulheres) - alterado
Massa do VE indexada<br>(g/m²)	<49 (homens) - alterado<br>49 – 115 (homens) - normalidade<br>>115 (homens) - alterado<br><br><43 (mulheres) - alterado<br>43 – 95 (mulheres) - normalidade<br>>95 (mulheres) - alterado
Espessura relativa da parede posterior	≤ 0,42 - normalidade<br>>0,42 - alterado
VSVE (mm)	<18 - alterado<br>18 – 22 (normalidade)<br>>22 - alterado
Veia cava inferior (mm)	≤ 21 - normalidade<br>>21 - alterado

- **Tabela B.** Termos pré-definidos no treinamento e captação do modelo de PLN para laudos ecocardiográficos e seus respectivos significados

Termo	Significado
ritmo cardíaco	Ritmo cardíaco observado e descrito pelo ecocardiografista
frequência cardíaca	Frequência cardíaca obtida durante o exame
movimento do septo interventricular	Descrição do movimento do septo interventricular
descrição vao	Descrição morfológica da valva aórtica
mobilidade vao	Avaliação da mobilidade da valva aórtica
prótese ao	Descrição morfológica, de mobilidade e refluxo da prótese aórtica
área vao	Medida da área valvar aórtica (se disponível)
descrição mi	Descrição morfológica da valva mitral
mobilidade mi	Avaliação da mobilidade da valva mitral
prótese mi	Descrição morfológica, de mobilidade e refluxo da prótese mitral
área mi	Medida da área valvar mitral (se disponível)
descrição tri	Descrição morfológica da valva tricúspide
mobilidade tri	Avaliação da mobilidade da valva tricúspide
prótese tri	Descrição morfológica, de mobilidade e refluxo da prótese tricúspide
gs max	Gradiente sistólico máximo da valva aórtica (se disponível)
gs med	Gradiente sistólico médio da valva aórtica (se disponível)
refluxo ao	Descrição do refluxo da valva aórtica
refluxo mi	Descrição do refluxo da valva mitral
refluxo tri	Descrição do refluxo da valva tricúspide
vsve	Medida do diâmetro da via de saída do ventrículo esquerdo (mm)
psap	Pressão sistólica da artéria pulmonar
derrame pericárdico	Descrição de derrame pericárdico, se houver
derrame pleural	Descrição de derrame pleural, se houver
veia cava inferior	Análise e medida da veia cava inferior
colabamento	Análise quanto à variação inspiratória da VCI
gd max	Gradiente diastólico máximo da valva mitral (se disponível)

gd med	Gradiente diastólico médio da valva mitral (se disponível)
contratilidade	Avaliação da contratilidade do VE (preservada *x* alteração de contratilidade segmentar)
função sistólica ve	Descrição da função sistólica do ventrículo esquerdo
função sistólica vd	Descrição da função sistólica do ventrículo direito
função sistólica biventricular	Descrição da função sistólica biventricular, se descrita dessa maneira no corpo do laudo
função diastólica ve	Descrição da função diastólica do ventrículo esquerdo
septo interatrial	Alterações morfofuncionais encontradas no septo interatrial
trombo	Descrição de trombo, se houver
vegetação	Descrição de vegetação, se houver
valva pulmonar	Descrição completa da valva pulmonar
altura	Altura do paciente no momento do exame
peso	Peso do paciente no momento do exame
asc	Área de superfície corpórea calculada no momento do exame
Raiz da aorta (seios de Valsalva)	Medida da aorta a nível dos seios de valsalva
Aorta ascendente proximal	Medida proximal da aorta ascendente
Átrio esquerdo (diâmetro)	Medida do diâmetro do átrio esquerdo
Átrio esquerdo (volume indexado)	Medida do volume indexado do átrio esquerdo
Diâmetro diastólico final do VE	Medida do diâmetro diastólico final do ventrículo esquerdo
Diâmetro sistólico final do VE	Medida do diâmetro sistólico final do ventrículo esquerdo
Espessura diastólica do septo	Medida da espessura diastólica do septo interventricular
Espessura diastólica da parede posterior	Medida da espessura diastólica da parede posterior
Fração de ejeção VE	Fração de ejeção do ventrículo esquerdo

Massa ventricular esquerda	Massa ventricular esquerda
Massa do VE indexada	Massa ventricular esquerda indexada
Espessura relativa da parede posterior	Espessura relativa da parede posterior
S’	Velocidade de deslocamento do anel tricúspide
Anel tricúspide	Medida do diâmetro do anel tricúspide (se disponível)
Aneurisma de VE	Descrição de aneurisma de ventrículo esquerdo, se houver

- **Tabela C.** Limitações e respectivas soluções na extração automática de dados

Limitação	Descrição	Solução
Lacunas em resultados laboratoriais	Foram extraídos apenas resultados da última coleta laboratorial (que em alguns indivíduos se tratava apenas do resultado de INR, isoladamente, por exemplo)	Preenchimento manual, a partir dos exames mais recentes de cada laboratorial de interesse
Obtenção do peso corporal	Não houve captação do valor do peso corporal em muitos pacientes, possivelmente por falha no preenchimento do prontuário	Preenchimento manual, a partir do peso incorporado no laudo de ecocardiograma utilizado
Obtenção do estágio de NYHA	Em alguns pacientes, houve lacuna no preenchimento automático, devido a preenchimento incompleto de prontuário	Preenchimento manual, a partir da descrição clínica presente na evolução médica mais recente
Preenchimento das colunas “IECA” e “BRA” (classes medicamentosas)	Em alguns pacientes, houve falha na captação e lacunas na tabela, devido a preenchimento incompleto (por não ter assinalado o “SIM” no campo específico às medicações, presente no prontuário	Preenchimento manual, a partir do nome das medicações presente no corpo do texto do prontuário
Etiologia da IT	A IA foi incapaz de descrever a etiologia da IT, por preenchimento incompleto de prontuário. É um dado que depende da interpretação médica, a	Preenchimento manual, a partir da interpretação feita pela autora do trabalho (por cada laudo de exame e informações clínicas de cada caso)

	partir do conjunto de achados no exame	

Tabela D. Padronização adotada para cálculo do TRI-SCORE

Idade > 70 anos	Coluna específica para idade
Sexo feminino	Coluna específica para sexo
NYHA III ou IV	Coluna específica para NYHA – pontua "SIM", se NYHA III ou IV
Sinais de IC direita	Coluna específica para edema e para turgência jugular – pontua "SIM", na presença de, no mínimo, uma das variáveis
Intervenção prévia	Coluna específica para essa informação – preenchida manualmente conforme relato de cirurgia prévia pelo prontuário
Marca-passo (dispositivo eletrônico implantável)	Coluna específica para essa informação – preenchida conforme assinalado em prontuário eletrônico ou na descrição ecocardiográfica da presença do dispositivo
Fibrilação ou Flutter atrial	Coluna específica para FA/Flutter atrial
Dose de furosemida > 125 mg/dia	Coluna específica para furosemida na dose diária correspondente
Taxa de filtração glomerular < 30 ml/min/1,73m²	Coluna específica para TFG – dado retirado do site de resultados laboratoriais (método CKD-EPI)
Bilirrubinas totais elevadas	Coluna específica para "bilirrubinas totais elevadas" – preenchido manualmente em "SIM" e "NÃO", a partir do valor de referência do laboratório
Fração de ejeção do VE < 60%	Coluna específica para FEVE
Disfunção ventricular direita	Coluna específica para "função ventricular direita" (classificada em

	PRESERVADA ou DISFUNÇÃO), que foi preenchida a partir da análise visual subjetiva e/ou de parâmetros quantitativos de FAC, S’ e TAPSE registrados pelo ecocardiografista
Etiologia da IT	Coluna específica para etiologia – preenchida manualmente

- **Tabela E.** Padronização adotada para cálculo do EuroSCORE II

Idade	Variável quantitativa contínua | valor a ser configurado na fórmula	Valor da idade retirado da tabela foi configurado na fórmula
Sexo	Variável dicotômica – *feminino* ou *masculino*	Sexo retirado da tabela foi configurado na fórmula
Doença pulmonar crônica	Variável dicotômica – *sim* ou *não* | Uso crônico de broncodilatador ou corticosteroide	Retirado da tabela e configurado na fórmula
Arteriopatia	Variável dicotômica – *sim* ou *não* | História de doença carotídea, ou DAOP, ou amputação, ou intervenção em aorta abdominal	Retirado da tabela e configurado na fórmula
Mobilidade reduzida	Variável dicotômica – *sim* ou *não* | Grave restrição de mobilidade por doença neurológica ou esquelética	Para fins de padronização, estabeleceu-se NÃO pontuar este item para nenhum dos indivíduos
Cirurgia cardíaca prévia	Variável dicotômica – *sim* ou *não* | Cirurgia cardíaca prévia que inclua abertura do pericárdio	Retirado da tabela e configurado na fórmula
Endocardite ativa	Variável dicotômica – *sim* ou *não* | Uso de antimicrobiano para endocardite infecciosa no momento da abordagem proposta	Para fins de padronização, estabeleceu-se NÃO pontuar este item para nenhum dos indivíduos
Estado crítico pré-operatório	Em uso de inotrópico ou BIA ou LRA anúrica ou morte súbita abortada por FV/TVSP	Para fins de padronização, estabeleceu-se NÃO pontuar este item para nenhum dos indivíduos
Função renal	Variável categórica ordinal | Opções: normal (ClCr > 85 ml/min/1,73m²), disfunção moderada (ClCr entre 50-85 ml/min/1,73m²), disfunção grave (ClCr <50 ml/min/1,73m²) e dialítico.	Retirado da tabela e configurado na fórmula
DM em uso de insulina	Variável dicotômica – *sim* ou *não*	Retirado da tabela e configurado na fórmula

Angina CCS 4	Variável dicotômica – *sim* ou *não*	Para fins de padronização, estabeleceu-se NÃO pontuar este item para nenhum dos indivíduos
Função de VE	Variável categórica ordinal | Opções: boa (FEVE >50%), função moderada (FEVE 31-50%), ruim (FEVE 21-30%), muito ruim (FEVE <30%).	Retirado da tabela e configurado na fórmula
IAM recente	Variável dicotômica – *sim* ou *não*	Para fins de padronização, estabeleceu-se NÃO pontuar este item para nenhum dos indivíduos
Hipertensão pulmonar	Variável categórica ordinal | Opções: ausente (PSAP até 30 mmHg, HP moderada (PSAP entre 31 e 55 mmHg), HP grave (PSAP > 55 mmHg)	Retirado da tabela e configurado na fórmula
NYHA	Variável categórica ordinal | Opções: I, II, III ou IV	Retirado da tabela e configurado na fórmula
Cirurgia na aorta torácica	Variável dicotômica – *sim* ou *não*	Para fins de padronização, estabeleceu-se NÃO pontuar este item para nenhum dos indivíduos
Caráter da cirurgia	Variável categórica | Opções: eletiva, de urgência, de emergência, de salvamento (ou resgate).	Para fins de padronização, estabeleceu-se “procedimento eletivo” a todos os indivíduos
Extensão da cirurgia	Variável categórica | Opções: revascularização isolada, não-revascularização isolada, 2 tipos de procedimento ou 3 tipos de procedimento	Para fins de padronização, estabeleceu-se “procedimento não-revascularização isolado” a todos os indivíduos

- **Tabela F.** Definições adotadas

**Valvopatia mitral associada:** nessa variável da tabela, foram considerados como "sim" todos os portadores de a) regurgitação mitral moderada ou importante e/ou b) de estenose mitral moderada ou importante e/ou c) prótese valvar mitral.
**Valvopatia aórtica associada:** nessa variável da tabela, foram considerados como "sim" todos os portadores de a) regurgitação aórtica moderada ou importante e/ou b) de estenose aórtica moderada ou importante e/ou c) prótese valvar aórtica.
**Função sistólica do ventrículo esquerdo:** foi considerada a classificação comumente utilizada pela Ecocardiografia[31], incluída nos laudos dos exames da instituição e acessada retrospectivamente, qual seja: a) função preservada: FEVE entre 52% e 72% (homem) ou FEVE entre 54% e 74% (mulher); b) disfunção discreta: FEVE entre 41% e 51% (homem) ou FEVE entre 41% e 53% (mulher); c) disfunção moderada: FEVE entre 30% e 40%; d) disfunção grave: FEVE abaixo de 30%.<br>Foi utilizada uma coluna "fração de ejeção do VE" na tabela, com o valor numérico encontrado no exame, para uso nos escores, que utilizam classificações próprias (vide item 3.7).
**Hipertensão pulmonar:** a definição baseou-se nos critérios ecocardiográficos atuais descritos por *Zamorano et al.* (2025), utilizando a estimativa da pressão sistólica da artéria pulmonar (PSAP) a partir da velocidade do jato de regurgitação tricúspide [30] (vide tabela A do material suplementar II). Para aplicabilidade do EuroSCORE II, que utiliza a classificação em HP moderada e HP grave para o cálculo (vide tabela E do material suplementar II), foi utilizada coluna específica baseada no valor numérico da PSAP.
**Doença pulmonar:** caracterizada pela presença de SAHOS, ou por diagnóstico já firmado de asma ou DPOC, ou ainda pelo uso contínuo de corticoides inalatórios ou broncodilatadores.
