# Supplementary material for: Aplicação da Inteligência Artificial para Detecção, Caracterização e Estratificação de Risco de Pacientes com Insuficiência Tricúspide Grave
Source: Arq Bras Cardiol. 2026 May 29;123(4):e20250743. [Article in Portuguese] doi: 10.36660/abc.20250743 (PMC13398803; doi:10.36660/abc.20250743)
Supplement: Translation

# Translation: Artificial Intelligence–Driven Detection, Characterization, and Risk Stratification in Patients with Severe Tricuspid Regurgitation

**DOI:** 10.36660/abc.20250743i

**Authors:** Luna Varela do Carmo, Auristela Isabel de Oliveira Ramos, Dorival Julio Della Togna, Eduardo da Silva Farias, Dimytri Alexandre de Alvim Siqueira

**Affiliations:** 1 São Paulo SP Brazil Instituto Dante Pazzanese de Cardiologia, São Paulo, SP – Brazil

**Keywords:** Heart Failure, Heart Valve Diseases, Tricuspid Valve Insufficiency, Artificial Intelligence

## Abstract

**Background:**

Tricuspid regurgitation (TR) is prevalent and challenging to manage. There is a high mortality rate associated with isolated surgery and late referral for intervention. Additionally, there has been a recent expansion of transcatheter therapies. In Brazil, there is a lack of data and validation of prognostic tools.

**Objective:**

To investigate and define clinical, laboratory, and echocardiographic data from patients with severe TR based on variables collected through the training of an artificial intelligence (AI) model.

**Methods:**

This observational, single-center, non-interventional, retrospective study was based on 71,911 echocardiographic reports performed between 2021 and 2024. A natural language processing (NLP) model was developed in Python to identify cases of severe TR, extract variables, and perform automated calculation of the TRI-SCORE and EuroSCORE II, which were subsequently reviewed. A two-tailed p-value < 0.05 was considered statistically significant.

**Results:**

A total of 803 patients with severe TR were identified. The mean age was 68.8 ± 13.5 years, and 64.1% were female. Hypertension (71.7%) and atrial fibrillation (69.4%) were the most prevalent comorbidities. Secondary etiology predominated (96.8%), mainly associated with mitral valve disease (68.5%). Surgical risk was high (medians: EuroSCORE II = 13.4%; TRI-SCORE = 7). Mortality was 17.1%. In the secondary mortality analysis, multivariable regression identified independent predictors, such as high-risk TRI-SCORE (relative risk [RR] 2.19; 95% CI 1.14-4.21) and increased creatinine (RR 3.13; 95% CI 1.91-5.14).

**Conclusions:**

In the largest Brazilian cohort of severe TR, constructed with the aid of AI, the disease demonstrated high clinical and prognostic complexity. The application of NLP to large echocardiographic databases proved to be efficient and may support multicenter studies.

## Introduction

Tricuspid regurgitation (TR) is one of the most frequent valvular diseases,^1^ reaching a prevalence of 4% in individuals > 75 years^2^ and being more common in women.^3^ In moderate to severe degrees, TR is associated with worse prognosis, regardless of comorbidities, pulmonary pressure levels, and right ventricular (RV) function.^4^ Among the factors associated with unfavorable outcomes in severe TR, advanced age,^5–6^ RV dysfunction,^6–8^ pulmonary hypertension,^7,9^ atrial fibrillation (AF),^5,10^ renal or hepatic dysfunction,^6^ and New York Heart Association (NYHA) functional classes III-IV stand out.^11,12^

In a recent study including 13,615 patients with aortic, mitral, and tricuspid valvular diseases, all-cause mortality was higher in the presence of TR (hazard ratio [HR] 2.74; 95% CI 2.24-3.37) compared with aortic valve disease (HR 1.62; 95% CI 1.44-1.82) or mitral valve disease (HR 1.25; 95% CI 1.09-1.44).^1^ Furthermore, significant TR concomitant with aortic stenosis is associated with a 1-year mortality of 21.3%, regardless of the treatment of aortic stenosis;^13^ in transcatheter aortic valve implantation, the presence of significant TR is associated with higher mortality, and its persistence after the procedure is associated with worse survival.^14^

Regarding risk stratification, traditional surgical risk scores were not developed to predict outcomes of isolated tricuspid valve intervention. The TRI-SCORE was proposed in the context of the expansion of catheter-based therapies.^12^ It has been suggested that TRI-SCORE has superior performance in predicting mortality after transcatheter edge-to-edge repair of the tricuspid valve, compared with EuroSCORE II and the STS Score.^15^

In Brazil, there remains a lack of national data on the prevalence, severity, and prognosis of TR. In a cross-sectional study including 645 patients with rheumatic valvular disease, a rate of 96.6% of associated functional TR was observed, with 12.8% of cases classified as moderate or severe.^16^ In parallel, advances in data science have made programming languages such as Python widely used in medicine,^17^ particularly for natural language processing (NLP) in the automated interpretation of clinical texts.

The aims of this study were to define the clinical, laboratory, and echocardiographic profile of patients with severe TR based on variables extracted by an NLP model, to classify them according to EuroSCORE II and TRI-SCORE, and to evaluate factors associated with mortality.

## Methods

### Study design and population

This observational, single-center, non-interventional, retrospective cohort study included patients with an echocardiographic diagnosis of severe TR at the Instituto Dante Pazzanese de Cardiologia (IDPC) between 2021 and 2024. In 2025, a retrospective review of institutional records was conducted to identify deaths.

The study was not designed to perform etiological stratification into mutually exclusive groups and included patients with severe TR regardless of etiology, the presence of concomitant mitral and/or aortic valve disease, or prior cardiac surgery; these associations were described solely for characterization purposes.

### Eligibility criteria

Individuals aged > 18 years with an echocardiographic diagnosis of severe TR were eligible for inclusion. Transesophageal echocardiographic reports, transthoracic examinations related to congenital heart disease, and studies performed for targeted diagnostic purposes that did not allow a standardized global assessment were excluded.

### Development of the natural language processing model

Based on the institution's standardized echocardiographic reporting model, named entities were defined for automated case identification (Figures 1 and 2). The typical textual structure of the reports was converted into operational patterns for detection, such as the excerpt "Tricuspid valve: (…). On Doppler, it shows severe regurgitation…", which was mapped to the combination of the terms "tricuspid reg_entity" and "severe," enabling identification of eligible cases.

**Figure 1:** Excerpt from the Label Studio platform, highlighting the terms. IVC: inferior vena cava; LA: left atrium; LV: left ventricle; PASP: pulmonary artery systolic pressure; RV: right ventricle.

Source: Prepared by the authors.

**Figure 2:** Excerpt from the Label Studio platform, highlighting the report body. FAC: fractional area change.

Source: Prepared by the authors.

The model was trained using 56 reports previously manually annotated on the Label Studio platform, employing a pipeline based on named entity recognition and sentence segmentation (spaCy/Python). After training, the model achieved an accuracy of 86% and was applied for patient selection and automated extraction of variables of interest.

The echocardiographic terms used in model training are detailed in Table B of Supplementary Material II. A similar approach was used for extracting laboratory data from the institutional platform and clinical information from the electronic medical record.

### Data collection and storage

Patients with at least one echocardiographic report documenting severe TR during the study period were included. For the analysis of variables of interest (Table A, Supplementary Material I), the most recent available examination was considered.

All cases identified by the AI model underwent manual review, confirming full compliance with the inclusion criteria.

Data were stored in the REDCap platform and subsequently exported to Microsoft Excel for organization, visualization, and analysis.

### Risk score calculation

The criteria and standardization procedures used for the automated calculation of EuroSCORE II and TRI-SCORE are described in Tables D and E of Supplementary Material II.

### Statistical analysis

Categorical variables were described as absolute and relative frequencies. Continuous variables were presented as mean ± standard deviation when normally distributed, or as median and interquartile range (Q1-Q3) when non-normally distributed. Minimum and maximum values were additionally reported in the tables of Supplementary Material I.

Initially, univariate analyses were performed to compare groups with and without death. For continuous variables, the Mann-Whitney *U* test was used, and for categorical variables, the chi-square test in contingency tables or, when appropriate, Fisher's exact test in 2×2 tables with expected frequency < 5.

Given the unavailability of exact time-to-death data, the outcome was analyzed within a fixed time window. Crude and adjusted relative risks, with 95% CIs, were estimated using logistic regression. Variable selection for the multivariable model followed a backward stepwise strategy, with a retention criterion of 10% and a significance level of 5%.

Statistical analyses were performed using Stata, version 19.

Before the analyses, extreme values were systematically reviewed based on the original echocardiographic reports. Values confirmed as valid were retained, whereas implausible or non-variable data were corrected or excluded.

### Ethical considerations

The study was conducted in accordance with the Declaration of Helsinki and Resolution No. 196/96 of the Brazilian National Health Council. The protocol was approved by the IDPC Human Research Ethics Committee via Plataforma Brasil (CAAE 82634424.0.0000.5462; approval No. 7.866.969).

## Results

### Sample construction

Between 2021 and 2024, the tool identified 71,911 echocardiographic reports related to acquired pathologies. Of these, 1,110 contained a diagnosis of severe TR. The sample selection flow is presented in Figure 3.

**Figure 3:** Flowchart of sample construction. AI: artificial intelligence; TR: tricuspid regurgitation.

Source: Prepared by the authors.

After applying eligibility criteria and manual review, 803 patients were included in the final analysis. Among them, 138 died at the institution, according to a retrospective review of medical records conducted in 2025.

Baseline characterization of the sample was performed, including clinical, laboratory, and echocardiographic variables of the overall population as well as univariate and multivariable analyses focused on the mortality outcome.

### Overall study population

The clinical, laboratory, and echocardiographic characteristics of the population are detailed in Tables B, C, and D of Supplementary Material I. The cohort included 803 patients with severe TR, predominantly composed of elderly women, with a mean age of 68.8 ± 13.5 years, and wide variability in body mass index.

Most patients were non-smokers, and alcohol use was reported by a minority of the sample.

### Clinical characteristics

The population presented a high burden of comorbidities, particularly hypertension, dyslipidemia, diabetes mellitus, and chronic kidney disease (Table 1). AF was present in approximately two-thirds of the cohort.

**Table 1:** Baseline characteristics of the sample

Characteristic		Total sample (n = 803)
Demographic data		
Age, years†		68.8 ± 13.5
Female sex, n (%)		515 (64.1)
BMI, kg/m^2^†		26.7 ± 5.4
**Lifestyle habits, n (%)**		
**Smoking**		
	Non-smoker	512 (63.8)
	Former smoker	251 (31.3)
	Current smoker	40 (5.0)
**Alcohol use**		
	Non-drinker	688 (85.7)
	Drinker	115 (14.3)
**Risk scores**		
**EuroSCORE II**‡		13.4 (8.1-21.3)
	Low, n (%)	43 (5.4)
	Intermediate, n (%)	155 (19.3)
	High, n (%)	605 (75.3)
**TRI-SCORE odds**‡		7 (6-9)
	Low, n (%)	51 (6.3)
	Intermediate, n (%)	145 (18.1)
	High, n (%)	607 (75.6)
**Clinical characteristics, n (%)**		
NYHA classes III-IV		127 (15.9)
Edema		62 (7.7)
Hypertension		576 (71.7)
Insulin therapy		42 (5.2)
Diabetes mellitus		199 (24.8)
AF		509 (69.4)
Pulmonary disease		132 (16.4)
Mitral valve disease		550 (68.5)
Aortic valve disease		183 (22.8)
Use of ACEi		271 (33.8)
Use of ARB		274 (34.1)
**Treatment, n (%)**		
**Use of furosemide**		610 (76.0)
	Dose > 125 mg/day	69 (8.6)
**Anticoagulant use**		512 (63.8)
	Warfarin	268 (33.4)
	Rivaroxaban	244 (30.4)
**Cardiovascular history, n (%)**		
Prior cardiac surgery		235 (29.3)
CIED		123 (15.3)
Mitral prosthesis		174 (21.7)
Aortic prosthesis		106 (13.2)
Tricuspid repair		34 (4.2)

† Values expressed as mean ± standard deviation.

‡ Values expressed as median (interquartile range). ACE: angiotensin-converting enzyme; AF: atrial fibrillation; ARB: angiotensin receptor blockers; BMI: body mass index; CIED: cardiac implantable electronic device; DM: diabetes mellitus; NYHA: New York Heart Association. EuroSCORE II categories: low risk (< 4%); intermediate risk (4%-8%); high risk (> 8%). TRI-SCORE categories: low risk (≤ 3 points); intermediate risk (4-5 points); high risk (≥ 6 points).

Source: Prepared by the authors.

Pulmonary hypertension was observed in most evaluated patients, with a relevant proportion presenting elevated pulmonary artery systolic pressure (PASP). From a clinical perspective, NYHA functional classes I-II predominated. In addition, some patients had implantable cardiac electronic devices and clinical signs of peripheral congestion (Table 1).

### Etiology of tricuspid regurgitation

Secondary TR was predominant (777/803; 96.8%), consistent with the functional phenotype of the cohort, whereas primary TR was identified in 26 patients (3.2%).

Among secondary cases, there was a frequent association with left-sided valvular diseases, mainly mitral (68.5%) and, to a lesser extent, aortic (22.8%). Among primary cases with defined etiology (n = 22), rheumatic causes predominated, followed by chordal rupture, Ebstein anomaly, valve cleft, and carcinoid syndrome.

### Medications in use

Regarding pharmacological treatment, most patients were receiving angiotensin-converting enzyme inhibitors or angiotensin receptor blockers. Oral anticoagulation, with warfarin or rivaroxaban, was widely used, although a smaller proportion of the cohort did not receive this therapy.

Loop diuretics were also frequently used, predominantly furosemide, including a subset of patients on high doses. In contrast, insulin use was less prevalent.

### Prior procedures and risk stratification

Among the 803 included patients, 235 (29.3%) had a history of prior cardiac surgery, most commonly mitral valve prosthesis (biological or mechanical), followed by aortic valve prosthesis and prior tricuspid repair.

Risk stratification revealed a cohort predominantly at high surgical risk according to EuroSCORE II, with elevated values in most patients and predominance of the high-risk category. Similar findings were observed with TRI-SCORE, both in its simple version and in the odds-standardized form, with approximately three-quarters of the population classified as high risk (Table 1).

### Laboratory characteristics

There was wide variability in laboratory parameters, which reflects different degrees of systemic involvement within the cohort (Table 2).

**Table 2:** Laboratory variables

Variable (unit)	Sample
Hematocrit, %†	37.5 (33.1-41.6)
Leukocytes, 10^3^/μL†	6.8 (5.4-8.7)
Platelets, cell/μL†	189,000 (147,000-236,000)
Creatinine, mg/dL†	1.3 (1.0-1.7)
GFR, mL/min/1,73 m^2^†	52 (35-72)
NT-proBNP, pg/mL†	2.517 (905-9.060)
Increased total bilirubin, n (%)	135 (17.3)

† Values expressed as median (interquartile range). GFR: glomerular filtration rate; NT-proBNP: N-terminal pro-B-type natriuretic peptide.

Source: Prepared by the authors.

Table 2 presents the median values of hematocrit, leukocytes, platelets, creatinine, glomerular filtration rate, and N-terminal pro-B-type natriuretic peptide (NT-proBNP). In addition, increased total bilirubin levels were identified in part of the patients.

### Echocardiographic characteristics

A complete description is provided in Table 3. Significant left atrial dilation was observed, associated with left ventricular (LV) remodeling and wide variability in systolic function, with reduced ejection fraction in more than half of the cohort.

**Table 3:** Echocardiographic variables

Variable	Sample
Secondary etiology, n (%)	777 (96.8)
IVC, mm†	23 (20-27)
Collapse < 50%, n (%)	536 (67.6)
Pericardial effusion, n (%)	143 (17.8)
PASP, mmHg†	53 (45-65)
RV dysfunction, n (%)	436 (54.3)
LVEF, %†	48 (31-58)
Significant LV dysfunction, n (%)	188 (23.4)
LV end-diastolic diameter, mm†	54 (48-60)
LV end-systolic diameter, mm†	38 (31-48)

† Values expressed as median (interquartile range). IVC: inferior vena cava; LV: left ventricle; LVEF: left ventricular ejection fraction; PASP: pulmonary artery systolic pressure; RV: right ventricle.

Source: Prepared by the authors.

RV systolic function was impaired in a similar proportion. PASP showed wide variation, with increased values in most patients (median 53 mmHg), often accompanied by inferior vena cava (IVC) dilation and reduced inspiratory collapse.

Pericardial effusion was identified in some cases. From a morphological standpoint, frequent valvular thickening was observed in the tricuspid, mitral, and aortic valves.

On the most recent available echocardiogram, most patients remained with severe TR. Among those who showed a reduction in severity, some had undergone surgical interventions, including tricuspid repair, heart transplantation, or left-sided valve surgery, whereas more than half remained under conservative management.

### Multivariable regression analysis

Variables identified in the univariate analysis or considered clinically relevant were included in the multivariable regression model.

In the final model (n = 763), adjusted using logistic regression with backward stepwise selection, independent predictors of mortality included high surgical risk according to TRI-SCORE, laboratory abnormalities indicative of organ dysfunction (decreased hematocrit, leukocytosis, thrombocytopenia, and increased creatinine), and echocardiographic findings associated with systemic congestion, such as IVC dilation and pericardial effusion.

Among structural variables, aortic valve thickening also remained associated with the outcome. Conversely, the use of rivaroxaban was independently associated with a lower probability of mortality (Table 4).

**Table 4:** Multivariable analysis

Variable	Category	RR	95% CI	p-value
TRI-SCORE	Low + intermediate	1.00	Reference	—
	High	2.19	1.14-4.21	0.019
Anticoagulant	No	1.00	Reference	—
	Warfarin	0.62	0.37-1.04	0.072
	Rivaroxaban	0.44	0.26-0.76	0.003
Hematocrit	Normal	1.00	Reference	—
	Abnormal	2.30	1.32-4.01	0.003
Leukocytes	Normal	1.00	Reference	—
	Abnormal	3.08	1.96-4.83	< 0.001
Platelets	Normal	1.00	Reference	—
	Abnormal	2.25	1.45-3.49	< 0.001
Creatinine	Normal	1.00	Reference	—
	Abnormal	3.13	1.91-5.14	< 0.001
Aortic valve thickening	No	1.00	Reference	—
	Yes	1.98	1.09-3.61	0.025
Pericardial effusion	No	1.00	Reference	—
	Yes	1.71	1.03-2.82	0.038
Inferior vena cava	Normal	1.00	Reference	—
	Abnormal	2.44	1.37-4.35	0.002

RR: relative risk.

Source: Prepared by the authors.

## Discussion

### Main findings and comparison with the literature

This cohort, comprising 803 patients with severe TR, represents, to date, the largest national case series on the subject. The construction of the sample was enabled through the application of artificial intelligence (AI), using an NLP model specifically developed for this study. This model allowed automated and standardized identification of reports compatible with severe TR from a dataset exceeding 70,000 echocardiographic examinations performed over 4 years at the institution.

This strategy enabled greater efficiency in case screening, reduced biases associated with manual data extraction, and improved reproducibility through systematic selection of included patients. All cases identified by the model as severe TR were confirmed after manual review of the echocardiographic reports, reinforcing the accuracy of the approach employed.

The study population was characterized by advanced age (mean 68.8 ± 13.5 years) and predominance of women (64.1%), a profile consistent with international registries such as the Framingham Heart Study,^3^ which demonstrated a higher prevalence of moderate to severe TR in elderly women, predominantly in the age range of 70–83 years. A high prevalence of comorbidities was observed, including hypertension, dyslipidemia, chronic kidney disease, and coronary artery disease, conditions common in this age group and associated with increased cardiovascular risk.

AF was present in 69.4% of patients, while approximately 43% had PASP ≥ 55 mmHg. The coexistence of these findings reflects relevant pathophysiological mechanisms in TR, including atrial dilation, volume overload, and the development of functional atrial TR as well as possible manifestations of more advanced disease stages.

The prevalence of AF observed was lower than that reported in clinical studies such as TRILUMINATE^18^ (90%) and TRISCEND II^19^ (96.1%), but similar to that described in TRIGISTRY^20^ (66%). Approximately 63% of the population was receiving oral anticoagulation, a proportion consistent with the prevalence of AF in the cohort.

Most patients were in NYHA functional classes I-II (84%), highlighting dissociation between the anatomical severity of TR and symptom burden. However, 76% were receiving diuretics, reflecting clinical management of systemic congestion. In contrast, more advanced clinical manifestations (e.g., NYHA class III-IV dyspnea [15.9%], use of furosemide at doses > 125 mg/day [8.6%], and lower limb edema [7.7%]) were less frequent, features that generally guide the indication for intervention.

Thus, in contemporary studies comparing percutaneous treatment strategies, inclusion criteria tend to favor patients with refractory symptoms, resulting in a higher proportion of individuals in more advanced functional classes. Reflecting this profile, the rate of diuretic use in our cohort was lower than that observed in other series of patients with severe TR.^18,20^ In addition, the possibility of clinical underestimation of symptoms or inaccuracies in medical records cannot be excluded, factors that may contribute to the discrepancy between reported functional status and the severity of tricuspid dysfunction.

The analysis of pharmacological treatment focused on the use of loop diuretics as an indirect marker of systemic congestion management, and comparison between different drug classes was not an objective of the study.

Secondary or functional TR was predominant in this cohort (97%). The high prevalence of associated left-sided valvular disease, especially mitral disease (68.5%), is consistent with prior research,^18–21^ which describes a frequent association between severe mitral regurgitation and severe TR, affecting approximately 30%-50% of such patients.

Approximately one-third of the population had undergone prior cardiac surgery, a proportion slightly lower than that reported in other series^18,22^ (29.3% vs 37% and 40%, respectively). The rate of prior valvular intervention involving the tricuspid valve, either isolated or concomitant, was low (4%), a finding consistent with previous literature.^23^

The proportion of secondary etiology observed exceeds traditional estimates, which indicate a prevalence of approximately 80% functional TR, 5%-10% primary TR, and about 10% associated with implantable cardiac electronic devices (CIEDs).^4,24^ This finding may be partially explained by the inclusion criteria adopted, which considered only acquired valvular diseases and excluded congenital forms, limiting the identification of primary etiologies, which accounted for 3.2% of cases. Approximately 15% of patients had CIEDs.

Laboratory findings reflect the systemic impact of severe TR, showing alterations consistent with chronic venous congestion. Decreases in hematocrit and platelet levels, together with increased levels of creatinine and bilirubin, suggest multisystem involvement. Increased neurohormonal markers, such as NT-proBNP, reinforce the presence of persistent hemodynamic overload and compensatory activation, features typical of patients with RV dysfunction and associated pulmonary hypertension.

After risk stratification using EuroSCORE II and TRI-SCORE, more than 75% of patients were classified as high surgical risk, highlighting the complex clinical profile of the cohort. This scenario suggests that, according to contemporary guidelines,^25^ transcatheter approaches may represent a relevant therapeutic alternative when anatomically feasible and available. Moreover, the proportion of patients classified as high risk by TRI-SCORE was substantially higher than that reported by Dreyfus et al.^20^ (75% vs 35%), which reinforces the greater clinical severity of this population.

Echocardiographic findings demonstrated structural and functional changes consistent with biventricular cardiac remodeling. LV end-systolic and end-diastolic diameters showed median values borderline to elevated for women but within normal limits for men, according to the cutoffs described in the supplementary material. LV end-diastolic diameter was similar to that observed in the TRISCEND II^19^ and TRILUMINATE^18^ studies (≈ 50-55 mm), which suggests mild LV remodeling.

LV ejection fraction (median 48%) was close to values reported in the TRIGISTRY^20^ registry and the EVOQUE TTVR^22^ (≈ 51%-54%), which indicates mild systolic dysfunction. Notably, 23.4% of patients had significant LV dysfunction, possibly related to prior valvular disease, interventricular interaction, and/or pulmonary hypertension.

RV dysfunction was present in 54.3% of patients, consistent with the range reported in multicenter cohorts (45%-60%), reinforcing the central role of right-sided chamber remodeling in the pathophysiology of severe TR. In addition, elevated PASP values (≈ 55 mmHg) corroborate the presence of chronic pressure overload, a key factor in the progression of RV dysfunction and perpetuation of TR.

In approximately 15% of patients, variation in TR severity was observed between the index echocardiogram used for study inclusion and the most recent examination from which variables were extracted. In these cases, the initial examination demonstrated severe TR, whereas subsequent evaluations showed a reduction in TR severity. Among patients with this variation, 41.4% underwent surgical intervention, including heart transplantation or left-sided surgery, with or without concomitant tricuspid valve intervention. Conversely, more than half remained under clinical management, suggesting that hemodynamic fluctuations and compensatory mechanisms may modulate TR severity over time.

Between the period of examination inclusion (2021-2024) and database lock (July 2025), when a retrospective review of institutional records was performed to identify outcomes, 138 patients (17.1%) died. This proportion corresponds to cumulative mortality within the fixed observation window adopted in the study and does not reflect individual time to event, as no systematic longitudinal follow-up was performed. The observed mortality rate was substantial and similar to that described in prior research,^26^ which reported approximately 20% mortality in patients with severe TR over a median follow-up period of 2.7 years.

To identify factors associated with mortality, an exploratory analysis including clinical, laboratory, and echocardiographic variables was performed. In the multivariable analysis, independent predictors of mortality included high TRI-SCORE, laboratory abnormalities (e.g., reduced hematocrit, leukocytosis, thrombocytopenia, and elevated creatinine), aortic valve thickening, IVC dilation, presence of pericardial effusion, and absence of rivaroxaban use.

Laboratory parameters maintained an independent association with the outcome, supporting their prognostic value. Reduced hematocrit may reflect anemia of chronic disease; thrombocytopenia may be related to hypersplenism or portal congestion; leukocytosis may indicate intermittent inflammatory or infectious processes; and increased creatinine suggests kidney dysfunction, often of congestive or multifactorial origin. The persistence of these variables in the adjusted model reinforces the importance of routine laboratory assessment as a prognostic tool, as described in recent studies and incorporated into contemporary scores such as the Isolated Tricuspid Valve Surgery Risk Calculator of the Society of Thoracic Surgeons (STS).^27^

Among echocardiographic variables, IVC dilation and pericardial effusion remained significantly associated with mortality, which reinforces the role of systemic venous congestion and right-sided chamber remodeling as markers of advanced disease.^28^ Increased IVC diameter is a well-established marker of increased right atrial pressure and volume overload, frequently described in severe TR.^29,30^ Pericardial effusion, in turn, may reflect hemodynamic decompensation and increased intracardiac pressures, often associated with pulmonary hypertension and RV dysfunction, even when small in magnitude.^31^

Aortic valve thickening also remained an independent predictor, suggesting that this finding, although not directly related to the tricuspid valve, may reflect systemic cardiovascular disease and diffuse valvular degeneration associated with aging, arterial stiffness, and increased atherosclerotic burden, features of patients at more advanced disease stages.

The use of rivaroxaban was independently associated with lower mortality. This effect may be related to reduction in thromboembolic events and a potential decrease in pulmonary microthrombosis, resulting in lower RV overload. In addition, favorable pharmacological characteristics, such as predictable pharmacokinetics and absence of international normalized ratio (INR) variability, may have contributed to the consistency of the observed effect. However, the influence of treatment allocation bias cannot be excluded, as direct oral anticoagulants tend to be prescribed to patients with nonvalvular AF and a less severe clinical profile.

TRI-SCORE emerged as the most robust predictor in the multivariable model, even after adjustment for multiple covariates, highlighting its integrated and multidimensional nature. In the final model, the score incorporated the effect of its component variables, reducing collinearity and establishing itself as an independent risk marker.

### Practical clinical implications

The findings of this study have relevant implications for the clinical management of patients with severe TR. In this large cohort, TRI-SCORE showed consistent prognostic performance and may contribute to risk stratification and therapeutic decision-making in the heart team setting. To the best of current knowledge, this is the first national study to systematically evaluate this score in a large population with severe TR, providing local evidence and initial parameters for its calibration and application in Brazilian patients.

Although the observational nature of the study requires caution regarding the broad adoption of TRI-SCORE, the results generate hypotheses that may, in the future, help guide follow-up protocols and selection criteria for intervention. In this regard, early risk stratification with TRI-SCORE, combined with periodic reassessment of its trajectory, may be useful in clinical practice. Patients initially classified as low risk may benefit from closer surveillance, considering the possibility of intervention at earlier stages of the disease, a strategy aligned with findings from multicenter registries that stratify outcomes based on this score.^20^

The association between laboratory markers (e.g., hematocrit, leukocytes, platelets, and creatinine) and mortality as well as between echocardiographic signs of right-sided congestion (IVC dilation with decreased inspiratory collapse) and pericardial effusion, reinforces the importance of longitudinal monitoring of these parameters. In practice, this means paying systematic attention to signs of organ dysfunction, especially in the kidneys, blood cells, and liver. These signs may precede adverse outcomes and serve as objective markers for discussing therapeutic intensification with the heart team early on.

The independent association between rivaroxaban use and lower mortality suggests that, beyond formal indications (e.g., AF), there may be differential effects related to greater pharmacokinetic predictability and lower INR variability in settings of liver congestion. However, this finding does not allow causal inference and should be interpreted with caution, especially given the possibility of treatment allocation bias.

### Limitations of study

The retrospective and single-center design exposes the study to potential selection biases and to the limitations inherent to the use of secondary data, including inconsistencies in medical record documentation and the absence of active patient follow-up. From a methodological perspective, automated data extraction through NLP had limitations, such as missing information for certain variables in part of the analyzed individuals (Table C, Supplementary Material II), which required active manual completion.

In addition, the adopted design was cross-sectional, based on the most recent available echocardiogram for variable analysis, and did not include longitudinal assessment of the patients' echocardiographic evolution.

Assessment of the mortality outcome was restricted to the institutional setting, which may have led to underestimation of events due to the absence of external records. The lack of information on time to death precluded survival analyses, and multivariable modeling with a binary outcome in a fixed time window was adopted. Furthermore, it was not possible to systematically distinguish between perioperative and late deaths, nor to identify loss to follow-up, due to the absence of longitudinal monitoring.

Finally, although therapeutic strategies are clinically relevant for severe TR, especially in patients with secondary etiology and a history of prior left-sided valve intervention, the study design did not allow comparison of outcomes according to the therapeutic approach adopted.

### Future perspectives

The findings of this study reinforce the need to expand data generation on TR in Brazil, particularly through multicenter studies that allow external validation of TRI-SCORE and comparative evaluation with other prognostic models, such as the STS Isolated Tricuspid Valve Surgery Risk Calculator.

Prospective studies will be essential to clarify the role of pharmacological interventions, especially anticoagulation, in the clinical outcomes of patients with severe TR.

In light of recent advances in transcatheter therapies, characterizing the risk profile of the Brazilian population becomes essential to optimize candidate selection and maximize therapeutic benefits. In this context, early identification of laboratory and echocardiographic markers associated with worse prognosis may favor the indication of intervention at less advanced stages of the disease, reducing the impact of late referral, which has historically been associated with worse outcomes.

## Conclusions

This study describes a large cohort of patients with severe TR identified through AI techniques applied to echocardiographic reports. The automated approach proved feasible and accurate, allowing comprehensive characterization of the clinical, laboratory, and echocardiographic aspects of a population predominantly composed of elderly women, with secondary etiology, a high burden of comorbidities, especially hypertension and AF, and frequent association with significant mitral valve disease and high-risk scores.

Risk stratification showed a high proportion of patients at elevated surgical risk, with TRI-SCORE emerging as an independent predictor of mortality, reinforcing the prognostic impact of severe TR and the complexity of its management.

Taken together, these findings highlight the potential of AI in building large cohorts, supporting predictive models, and optimizing risk stratification strategies, thereby contributing to a more precise definition of the ideal timing for valvular intervention (Central Illustration).

## Data Availability

The underlying content of the research text is contained within the manuscript.

## *Supplemental Materials

For Supplementary Material I, please click here.

For Supplementary Material II, please click here.
